# Use of behavior change techniques in physical activity programs and services for older adults: findings from a rapid review

**DOI:** 10.1093/abm/kaad074

**Published:** 2024-02-01

**Authors:** Heidi Gilchrist, Juliana S Oliveira, Wing S Kwok, Catherine Sherrington, Marina B Pinheiro, Adrian Bauman, Anne Tiedemann, Leanne Hassett

**Affiliations:** Sydney School of Public Health, Faculty of Medicine and Health, Sydney Musculoskeletal Health, University of Sydney, Gadigal Country, Sydney, Australia; Institute for Musculoskeletal Health, University of Sydney and Sydney Local Health District, Gadigal Country, Sydney, Australia; Sydney School of Public Health, Faculty of Medicine and Health, Sydney Musculoskeletal Health, University of Sydney, Gadigal Country, Sydney, Australia; Institute for Musculoskeletal Health, University of Sydney and Sydney Local Health District, Gadigal Country, Sydney, Australia; Sydney School of Public Health, Faculty of Medicine and Health, Sydney Musculoskeletal Health, University of Sydney, Gadigal Country, Sydney, Australia; Institute for Musculoskeletal Health, University of Sydney and Sydney Local Health District, Gadigal Country, Sydney, Australia; Sydney School of Public Health, Faculty of Medicine and Health, Sydney Musculoskeletal Health, University of Sydney, Gadigal Country, Sydney, Australia; Institute for Musculoskeletal Health, University of Sydney and Sydney Local Health District, Gadigal Country, Sydney, Australia; WHO Collaborating Centre for Physical Activity, Nutrition and Obesity, Charles Perkins Centre, University of Sydney, Gadigal Country, Sydney, Australia; Sydney School of Public Health, Faculty of Medicine and Health, Sydney Musculoskeletal Health, University of Sydney, Gadigal Country, Sydney, Australia; Institute for Musculoskeletal Health, University of Sydney and Sydney Local Health District, Gadigal Country, Sydney, Australia; Sydney School of Public Health, Faculty of Medicine and Health, Sydney Musculoskeletal Health, University of Sydney, Gadigal Country, Sydney, Australia; WHO Collaborating Centre for Physical Activity, Nutrition and Obesity, Charles Perkins Centre, University of Sydney, Gadigal Country, Sydney, Australia; Sydney School of Public Health, Faculty of Medicine and Health, Sydney Musculoskeletal Health, University of Sydney, Gadigal Country, Sydney, Australia; Institute for Musculoskeletal Health, University of Sydney and Sydney Local Health District, Gadigal Country, Sydney, Australia; WHO Collaborating Centre for Physical Activity, Nutrition and Obesity, Charles Perkins Centre, University of Sydney, Gadigal Country, Sydney, Australia; Institute for Musculoskeletal Health, University of Sydney and Sydney Local Health District, Gadigal Country, Sydney, Australia; Sydney School of Health Sciences, Faculty of Medicine and Health, Sydney Musculoskeletal Health, University of Sydney, Gadigal Country, Sydney, Australia

**Keywords:** Aged, Behavior change techniques, Exercise, Health behavior, Intervention studies, Physical activity

## Abstract

**Background:**

Understanding behavior change techniques (BCTs) used in randomized controlled trials (RCTs) of physical activity programs/services for older adults can help us to guide their implementation in real-world settings.

**Purpose:**

This study aims to: (a) identify the number and type of BCTs used in physical activity programs/services for older adults evaluated in large, good quality RCTs and (b) explore the impact of different BCTs on different outcome domains.

**Methods:**

This is a secondary data analysis of a WHO-commissioned rapid review of physical activity programs/services for older adults. Fifty-six trials testing 70 interventions were coded for the type and number of BCTs present using a published BCT taxonomy. The proportion of positive effects found from physical activity interventions using the most common BCTs was calculated for the outcomes of physical activity, intrinsic capacity, functional ability, social domain, cognitive and emotional functioning, and well-being and quality of life.

**Results:**

Thirty-nine of the 93 possible BCTs were identified in the included trials and 529 BCTs in total (mean 7.6, range 2–17). The most common BCTs were “action planning” (68/70 interventions), “instructions on how to perform a behavior” (60/70), “graded tasks” (53/70), “demonstration of behavior” (44/70), and “behavioral practice/rehearsal” (43/70). Interventions that used any of the most common BCTs showed overwhelmingly positive impacts on physical activity and social domain outcomes.

**Conclusion:**

Consideration of which BCTs are included in interventions and their impact on outcomes can improve the effectiveness and implementation of future interventions. To enable this, providers can design, implement, and evaluate interventions using a BCT taxonomy.

## Teaser

Using behaviour change techniques (BCTs) in physical activity interventions for older adults improves physical activity outcomes, and different BCTs impact other outcomes such as physical capacity, functional ability, wellbeing and quality of life. Accurate reporting of BCTs and consideration of the desired outcome is essential to maximise effectiveness of future interventions

## Introduction

It is well established that regular physical activity can help prevent and manage noncommunicable diseases [1, 2], improve mental health and well-being, and maintain a healthy weight [3]. In adults aged over 60 years, regular physical activity can also prevent falls, maintain physical function, and delay the onset of dementia [2, 4]. Consequently, the 2020 World Health Organization (WHO) guidelines on Physical Activity and Sedentary Behaviour recommend regular moderate to vigorous aerobic physical activity for older adults as well as exercises targeting strength and balance/function [2]. However, recent research shows that globally more than a quarter of all adults, and close to half of those aged over 70 years, are insufficiently active, and this number has increased rather than declined in the past decade [5, 6]. The number of adults aged over 60 years is also predicted to double in the next three decades [6]. Identifying and implementing programs and strategies that effectively increase physical activity and improve the functional abilities of the older population should be a priority [7].

The WHO Global Action Plan on Physical Activity (GAPPA) describes four strategic objectives (Active Societies, Active Environments, Active People, Active Systems) achievable through 20 policy actions to address global inactivity. To facilitate GAPPA’s implementation, the WHO is creating technical toolkits with implementation guidance for key approaches, settings, and populations [8]. Our research group conducted a scoping review of systematic reviews of physical activity interventions for older adults to inform the development of the toolkit on physical activity for older adults [9]. The mixed nature of the settings, populations, and interventions of the studies included in the reviews made specific recommendations difficult. Therefore, we undertook a further rapid review, examining individual studies, to inform the WHO and others regarding the effectiveness of physical activity programs and services for older adults on various outcomes. Specifically, the rapid review classified the types of physical activity interventions in individual studies, that is, physical activity promotion, structured exercise, recreation, and sport (collectively these interventions have been defined in the rapid review as programs or services) as well as different subpopulations (e.g., people with low mood or impaired cognition) and different locations (e.g., community settings or health facilities) using a framework designed for the purpose (and described in detail elsewhere) [10]. The effect of the above components on seven different outcome domains, compared with no intervention, was then examined. The outcome domains identified were physical activity (e.g., steps, overall physical activity), falls, intrinsic capacity: physical domain (e.g., pain, bone mineral density and strength) [11, 12], functional ability in three areas—physical (e.g., balance and mobility, overall function), social (self-report measures, which are defined as “participation” by the WHO’s International Classification of Functioning, Disability and Health or ICF [13]), cognitive and emotional (either or both) and well-being and quality of life (QoL). These domains and examples of measures within these domains can be seen in the table of study characteristics in Supplementary Appendix 5.

This rapid review of individual studies reported the results of 87 good quality (PEDro score of 6 or more [14]) randomised controlled trials (RCTs) investigating the effects of physical activity programs and services for older adults compared with no intervention, categorized according to population and intervention characteristics. The identified trials had overwhelmingly positive findings in favor of physical activity (>75% of physical activity outcomes were positive) [15].

Interventions designed to change health-related behaviors such as physical activity are often complex and involve numerous components to support a participant in the change process [16]. It is important to be able to accurately identify and describe the “active ingredients,” including behavior change techniques (BCTs), of physical activity programs tested in trials so they can be compared, and successful program components replicated [17, 18], and included when the program or service is implemented in practice. Previous systematic reviews of physical activity interventions have identified and described BCTs used in each study [16, 19–28], and considered the relationship of BCTs with study outcomes. Most of these reviews, however, were conducted in the general adult populations, sometimes with specific characteristics (e.g., cancer survivors). Older people have unique needs, with different physical, emotional, and cognitive abilities as well as changing financial and environmental circumstances [29], so different BCTs to those used in younger populations may be needed to effect physical activity behavior change. The few reviews that focused on older adults [21, 22, 30, 31] are at least 5 years old and provide contradictory evidence regarding which BCTs to include when designing physical activity interventions for the older population. For example, while goal setting (behavior) was identified as a promising strategy in one study [21], another found it to be associated with lower levels of physical activity and self-efficacy [22]. Additionally, some of these reviews use narrative analysis, and/or draw on within-group (i.e., the change within one group’s outcome measures from before to after the intervention) as well as between-group differences (i.e., the change between the intervention and the control group) [21, 25, 27, 30, 31]. In RCTs, within-group differences may be influenced by other factors (e.g., self-report bias). While between-group comparisons are not completely immune from bias, the use of good quality trial data (i.e., PEDro score of 7 or more) means that the between-group differences for these studies are more likely to give a true representation of the change in various outcomes due to the intervention. Furthermore, these studies have only considered one or two outcome measures, such as physical activity or adherence. Our rapid review demonstrated that different types of interventions impacted different outcome measures differently (e.g., for recreation/sport, the strongest impact was on cognitive and emotional functioning). Equally, there is a need to consider whether interventions that employ different BCTs have the same or different effects on various outcome measures, particularly as not all interventions’ sole purpose is to increase physical activity, for example, some aim to address social isolation or cognitive health. When different interventions are equally successful in promoting physical activity but have different impacts on other outcomes, it is possible that different BCTs may play a role as BCTs are not unique to only one outcome. For example, using a BCT from the grouping “social support” may improve both physical activity and social well-being (either directly through increased social contact, or indirectly through the benefits of physical activity) but a different intervention, which employs the BCT “graded tasks” may be equally successful at improving physical activity, but have less or no impact on social outcomes.

This review therefore seeks to contribute to the body of evidence by describing the BCTs included in large, good quality trials of physical activity programs and services for older adults and classifying them using common language from a published taxonomy of BCTs, the Behaviour Change Technique Taxonomy (BCTT, version 1). The BCTT is a reliable taxonomy that consists of 93 unique BCTs that are categorized into 16 hierarchical clusters (known as BCT groupings) [17, 32]. Each BCT has its own number identifying its group and position within that group and they are observable and replicable when designing and reporting interventions and intervention content.

As BCTs are often used together to maximize behavior change, some studies have used meta-regression to identify certain combinations of BCTs that may be more effective collectively in increasing physical activity, but the patterns identified are not consistent between studies [16, 33, 34]. Others have also criticized the methodology of these studies and subsequently shown no evidence that BCT clustering in physical activity interventions increases physical activity self-efficacy (a correlate of physical activity) other than those groupings used in the BCT Taxonomy described below [35]. We therefore chose to focus on the classification of BCTs and BCT groupings found in the large-scale, good quality RCTs for physical activity and older people identified in our review as this will strengthen the scientific basis for implementing effective physical activity interventions to address a range of outcomes in the older population.

## Objectives

This study reviewed the trials included in our recent rapid review [15, 36] to

1. identify and summarize the number and type of BCTs used in the different types of physical activity programs and services for older adults evaluated in the included trials.
2. describe the impact of interventions using common BCTs, BCT groupings, and different numbers of BCTs compared with no intervention on outcomes of physical activity, intrinsic capacity (physical domain), functional ability (physical domain), social domain, cognitive and emotional functioning, and well-being and QoL, in trials of physical activity programs and services for older adults.

## Method

This study is a secondary analysis of a rapid review of primary studies prepared for the WHO [36] that reviewed the effectiveness of physical activity programs and services for adults aged 60 years and above.

### Study selection

Our rapid review [36] searched for primary studies that were identified by screening systematic reviews investigating physical activity interventions for older adults from an earlier scoping review [9]. The initial search was conducted for the dates January 1, 2010, to November 1, 2020, on four databases (PEDro, MEDLINE, CINAHL, and the Cochrane Database of Systematic Reviews) and was updated on March 20, 2021, using the same search strategy and databases (see Supplementary Appendix 1 for eligibility criteria and search strategies for both the scoping and rapid reviews). Hand searching and an additional search incorporating terms related to sports were also completed (see Supplementary Appendix 2 for search strategies for the selection of primary studies investigating sports in older adults). All individual studies identified in this search were then titled and abstract were reviewed by a pool of eight reviewers. All reviewers received training on the eligibility criteria and regular meetings were conducted to discuss questions regarding the criteria. A second experienced reviewer from the pool checked the eligibility assessment of a randomly selected sample of studies (5%). Any disagreements were discussed and resolved, before being full text reviewed for final inclusion by two independent reviewers using the below inclusion criteria (see also Supplementary Appendix 4).

(a) Population: adults aged 60 years or older
(b) Intervention: physical activity program or service
(c) Comparison: no active intervention
(d) Outcome: physical activity, falls, intrinsic capability (physical domain), functional ability (physical, social and cognitive, and emotional domains), and well-being and QoL
(e) Study design: randomized controlled trial (RCT)
(f) Sample size: at least 50 participants per group
(g) Methodological quality: good quality, defined here as a score of at least 6 on the PEDro quality scale (a 0 to 10 scale consisting of 11 items used to assess key aspects of study quality, e.g., blinding, intention to treat, etc. [14])
(h) Results reported for between-group statistical comparisons for relevant outcomes

### Data extraction

#### Rapid review

The WHO report extracted data for 106 intervention groups from 107 papers, reporting on 87 good quality (PEDro score ≥6), large (≥50 participants per group) RCTs investigating the effects of physical activity programs and services for older adults compared with no intervention (see Supplementary Appendix 3 for flow chart of literature search). These 106 intervention groups were categorized according to population and intervention characteristics (including whether the intervention included components to support behavior change) using a well-piloted framework [15, 36]. Briefly, no studies included in the review were undertaken in low-income countries. No studies focused on diverse or underserved cultural or socioeconomic backgrounds and none were conducted with rural or remote populations. Outcomes were categorized as physical activity, falls, intrinsic capacity (physical domain), functional ability (physical domain), social domain, cognitive and emotional functioning, and well-being and QoL. These characteristics are detailed in Supplementary Appendix 5. For each outcome, the intervention effect was extracted and categorized as positive significant, positive nonsignificant, no/negligible effect (i.e., *p* = .000), negative nonsignificant, negative significant (a summary of these interventions, and their outcomes, by location where the intervention was conducted, can be found in Supplementary Appendix 5). This method is recommended when meta-analysis is not possible or feasible (e.g., due to heterogeneity of measures making getting a standardized effect size difficult) [37] and proportions of positive outcomes rather than the size of the effect can be used in order to capture the large breadth of programs, settings, and outcomes within a restricted time frame. Using only statistical significance is problematic as many trials will be underpowered so potentially important effects that do not reach statistical significance could be missed. We acknowledge, however, that combining statistically significant and statistically nonsignificant positive effects may also affect the results.

#### This study

To avoid duplication of work and to save time, studies with the primary outcome of falls were omitted from this analysis (31/87 trials) as they were included in a concurrent review of BCTs in trials with falls as the primary outcome being conducted by colleagues [38]. Therefore, data for 70 interventions from 56 RCTs which included the outcome measures of physical activity, intrinsic capacity (physical domain), functional ability (physical, social, or cognitive and emotional domains), and well-being and QoL were included in this study (see Supplementary Appendix 10 for a reference list of included studies).

Intervention components related to the use of BCTs and deemed to facilitate a change in the target behavior (i.e., physical activity) for the target population (i.e., people 60 years and older), which were not present in the control groups, were extracted and coded according to the BCTT [32] (see Supplementary Appendix 4). In 34 instances, protocol papers, supplementary reports, and other papers about the same study were available and were reviewed and assessed for additional BCTs. The BCTs identified were also categorized in the 16 groupings listed in the BCTT (see Supplementary Appendix 8 for frequency of BCTs in each grouping and Supplementary Appendix 6 for a full list of BCTs and groupings) and if an intervention contained one or more BCTs belonging to a grouping, it was coded to that grouping. Pairs of reviewers (HG, WSK, JSO, LH) independently coded the BCTs in 14 studies. All the reviewers had previous experience and/or had completed certified training in advance (available at www.bct-taxonomy.com). Disagreement was resolved through discussion and/or by the third review author if necessary. High (86.4%) agreement was found between reviewers, so one review author (HG) coded the remaining studies but consulted other authors when clarification or discussion was required.

### Data synthesis and analysis

To address objective one, the total number of BCTs used and the most frequent BCTs and BCT groupings used in physical activity interventions were examined as a whole and for different intervention characteristics.

For objective two, descriptive statistics were used to describe the impact of interventions with different BCT groupings, individual BCTs, and number of BCTs in each study on the outcomes of physical activity, intrinsic capacity (physical domain), functional ability (physical, social, or cognitive and emotional domains), and well-being and QoL. For each of these six outcome categories, the total number of outcomes, as well as the number of positive outcomes and the number of significant positive outcomes in each category, was extracted from the rapid review. We then calculated the number of overall positive outcomes (indicated by more than 50% of comparisons being in a positive direction) and positive significant outcomes in the trials that included the most common BCTs, BCT groupings, and certain numbers of BCTs for each outcome. These are presented in figures and described in subsequently.

## Results

### Use of BCTs

**Fig. 1.**
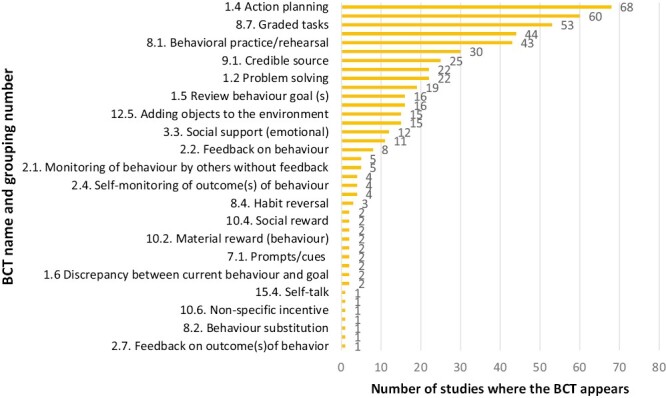
Number of times each identified BCT was used in PA programs and services for older people evaluated in the included trials

The 70 interventions analyzed contained a total of 529 BCTs (mean 7.6, range 2–17). Thirty-nine of 93 possible BCTs (42%) in the taxonomy were identified in the trials (see Fig. 1), meaning that 54 BCTs were not present in any of the trials. Of these 39 BCTs, some were much more commonly used than others. Five of the BCTs accounted for 51% (268/529) of all BCTs used and 10 BCTs accounted for 73% (386/529) of all BCTs used. The most common BCT was “action planning” (reported in 68/70 or 97% of interventions), followed by “instructions on how to perform a behavior” (60/70 or 85% of interventions), “graded tasks” (53/70 or 76% of interventions), “demonstration of behavior” (44/70 or 63% of interventions), and “behavioral practice/rehearsal” (43/70 or 61% of interventions).

Table 1 describes the intervention characteristics. For example, the most common type of intervention used was structured exercise (36/70, 51%), while recreation was less common (13/70, 19%). Interventions most often took place in the community (29/70, 41%), although 12/70 (17%) took place in the participant’s home. This table also demonstrates that some types of interventions used more BCTs on average than others. For example, interventions promoting increasing overall physical activity used an average of 9.7 BCTs per intervention, while interventions providing recreational physical activity used an average of 5.2 BCTs. Similarly, interventions carried out in the participants’ own homes used 9.1 BCTs on average, while those conducted in a residential aged care facility used 5.9 BCTs on average.

**Table 1:** Number of BCTs Used in the Studies of Physical Activity Programs and Services by Different Study Characteristics.

Study characteristic	BCTs total (mean ± std. dev.)
Type of physical activity	
Structured exercise (36 interventions)	261 (7.3 ± 3.1)
Promoting overall activity (19 interventions)	184 (9.7 ± 2.8)
Recreation (13 interventions)	68 (5.2 ± 1.0)
Competitive sport (1 intervention)	8 (8)
Exercise based video game (1 intervention)	8 (8)
Primary location of intervention	
Community (29 interventions)	186 (6.4 ± 2.0)
Own home (12 interventions)	109 (9.1 ± 3.1)
Health facility (4 interventions)	26 (6.5 ± 6.1)
Program promoted physical activity rather than delivering it (16 interventions)	156 (9.8 ± 3.1)
Residential aged care facility (8 interventions)	47 (5.9 ± 0.8)
Retirement village (1 intervention)	5 (5)
Physical activity as outcome	
Measured (25 interventions)	229 (9.2 ± 3.4)
Not measured (45 interventions)	300 (6.7 ± 3.1)

### Use of different BCT groupings

When considering the BCTs within their 16 BCTT taxonomy groupings, only 13 of the 16 groupings were represented. The most common BCT groupings were “goals and planning” and “substitution and repetition,” accounting for nearly half of the BCTs (247/529 BCTs or 47%). “Shaping knowledge,” “social support,” and “comparison of behavior” were the next most commonly used groupings (60, 53, and 51 BCTs, respectively) (see Supplementary Appendix 8). The three BCT groupings not used were “covert learning,” “scheduled consequences,” and “regulation.”

### Intervention effects by individual BCTs and outcome domain

**Fig. 2.**
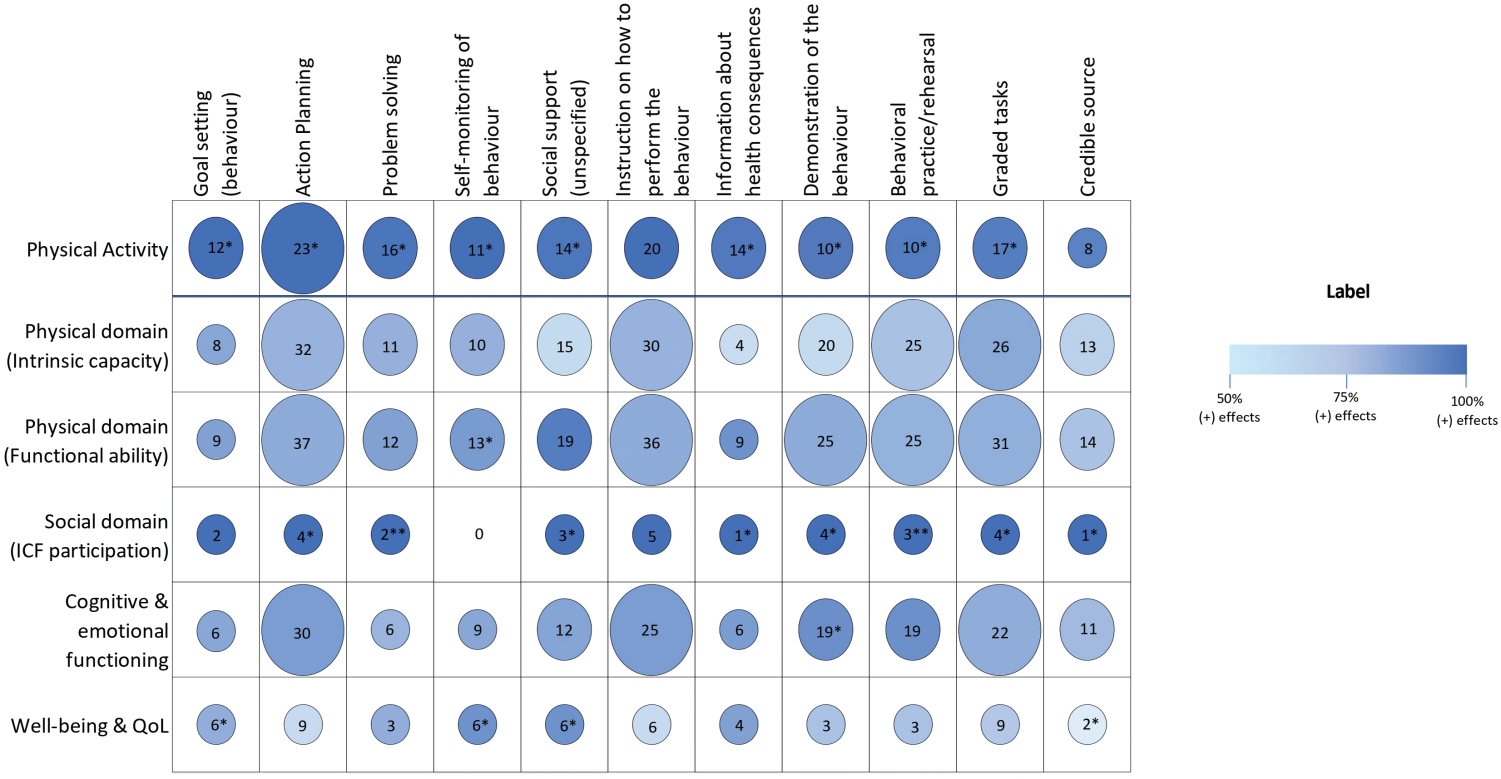
Intervention effects by the most common BCTs and outcome domain. The figure under Label presents the effect direction and statistical significance for outcome domains across primary studies. The numbers in each cell indicate the number of intervention groups. All intervention arms from the same study were included as a unique intervention group. The size of the circle depicts the number of intervention groups such that the larger the circle, the greater the number of intervention groups. Large circles represent ≥20 intervention groups; medium circles represent 10–20 intervention groups; and small circles represent <10 intervention groups. Shades of color depict the overall positive effect direction such that the darker the color, the higher the proportion of positive effects across comparisons. No shading reflects <50% positive effect or negative effects (there are none in this figure). *If 50 to 75% of total outcomes were reported as positive and statistically significant. For example, for the goals and physical activity outcomes circle, * indicates that 6–9 of 12 of the interventions tested which included the BCT goal setting had a positive and significant effect on physical activity outcome compared to their comparison no intervention groups. **If >75% of total outcomes were reported as positive and statistically significant.

Figure 2 shows the effect of interventions that included the most common BCTs, plus goal setting which was identified as an effective BCT in physical activity interventions by other systematic reviews [16, 21, 39], by outcome. Figure 2 also indicates the percent of between-group comparisons with overall positive effects, as well as positive and significant effects by outcome for each of these 11 BCTs (data used to create this figure are located in Table A.7.1 in Supplementary Appendix 7).

For physical activity outcomes, all interventions that used any of the examined BCT categories showed overwhelmingly positive impacts compared with no intervention, with more than 90% of overall positive effects for each BCT. Only the BCTs’ “instructions on how to perform a behavior” and “credible source” had less than 50% positive and significant overall effects. Similarly, 100% of social domain outcomes showed an overall positive effect for interventions that included any of the BCTs listed (although this included only between 0 and 5 studies, indicated by the small circles in Fig. 2) and only “instructions on how to perform a behavior” and “goal setting” had less than 50% positive and significant overall effects. Fewer overall positive effects were seen in the intrinsic capacity domain outcome and the well-being and QoL domain outcome. For the domain of intrinsic capacity, interventions that included “goal setting” and “graded tasks” showed more than 75% of overall positive effects. For well-being and QoL outcomes, only interventions with three of the examined BCTs (“self-monitoring of behavior,” “social support (unspecified),” and “information about health consequences”) showed more than 75% of overall positive effects. Interventions including the other BCTs had between 50 and 75% of overall positive effects on intrinsic capacity and well-being/QoL outcomes. For the physical domain (functional ability) interventions that included all examined BCTs except “behavioral practice/rehearsal,” “graded tasks,” or “credible source” showed greater than 75% overall positive effects. For the cognitive and emotional well-being domain, interventions that included any of the examined BCTs, except “problem solving,” showed greater than 75% overall positive effects.

Looking at the BCTs across all outcomes, only interventions that included “goal setting” had 75% or more overall positive effects for all domains (and two domains for goal setting had 50% or more positive and significant effects), although approaching this were “problem solving” (72%–100% of positive effects across all domains) and “self-monitoring of behavior” (74%–100% of overall positive effects across all domains).

### Intervention effects by BCT groupings and outcome domain

The impact of interventions containing the 11 most frequently appearing BCT groupings by outcome was also considered (see Table A.7.2 Supplementary Appendix 7 for data and Supplementary Appendix 9 for figure representation). The proportion of interventions with positive effects (and positive and significant effects) in each BCT grouping varied by outcome domain. Like the individual BCTs considered above, all interventions that used any of the BCT groupings showed overwhelmingly positive impacts in the physical activity domain (>80% overall positive effects) and social domain (100% overall positive effects). All but one grouping in the physical activity domain and all but two groupings in the social domain had 50% or greater positive and significant effects. On the other hand, for outcomes in the intrinsic capacity domain, only interventions that contained BCTs in the grouping “antecedents” had greater than 75% of overall positive effects, and interventions that contained any of the other 10 BCT groupings had between 50% and 75% of overall positive effects.

The other domains fell in between these extremes. For interventions with well-being and QoL domain outcomes, 6 of the 10 BCT groupings showed between 50% and 75% of overall positive effects. Four BCT groupings had >75% of overall positive effects—“feedback and monitoring,” “social support,” “natural consequences,” and “reward and threat.” For the cognitive and emotional functioning domain and the functional ability domain, interventions with 2 of the 11 BCT groupings also had between 50% and 75% of overall positive effects (effects for other BCT groupings were greater than 75% of overall positive effects).

Looking across all outcomes for BCT groupings, while none had more than 75% positive effects for all outcomes, some approached this, such as “goals and planning” (67%–100% range of overall positive effect), “feedback and monitoring” (70%–100% range of overall positive effect), “social support” (69%–100% range of overall positive effect), and “repetition and substitution” (67%–100% range of overall positive effect). Groupings with less overall positive effects were “comparison of outcomes” and “identity” (although there are only a small number of comparisons in each of these groupings across all outcomes).

### Intervention effects by number of BCTs used and outcome domains

**Fig. 3.**
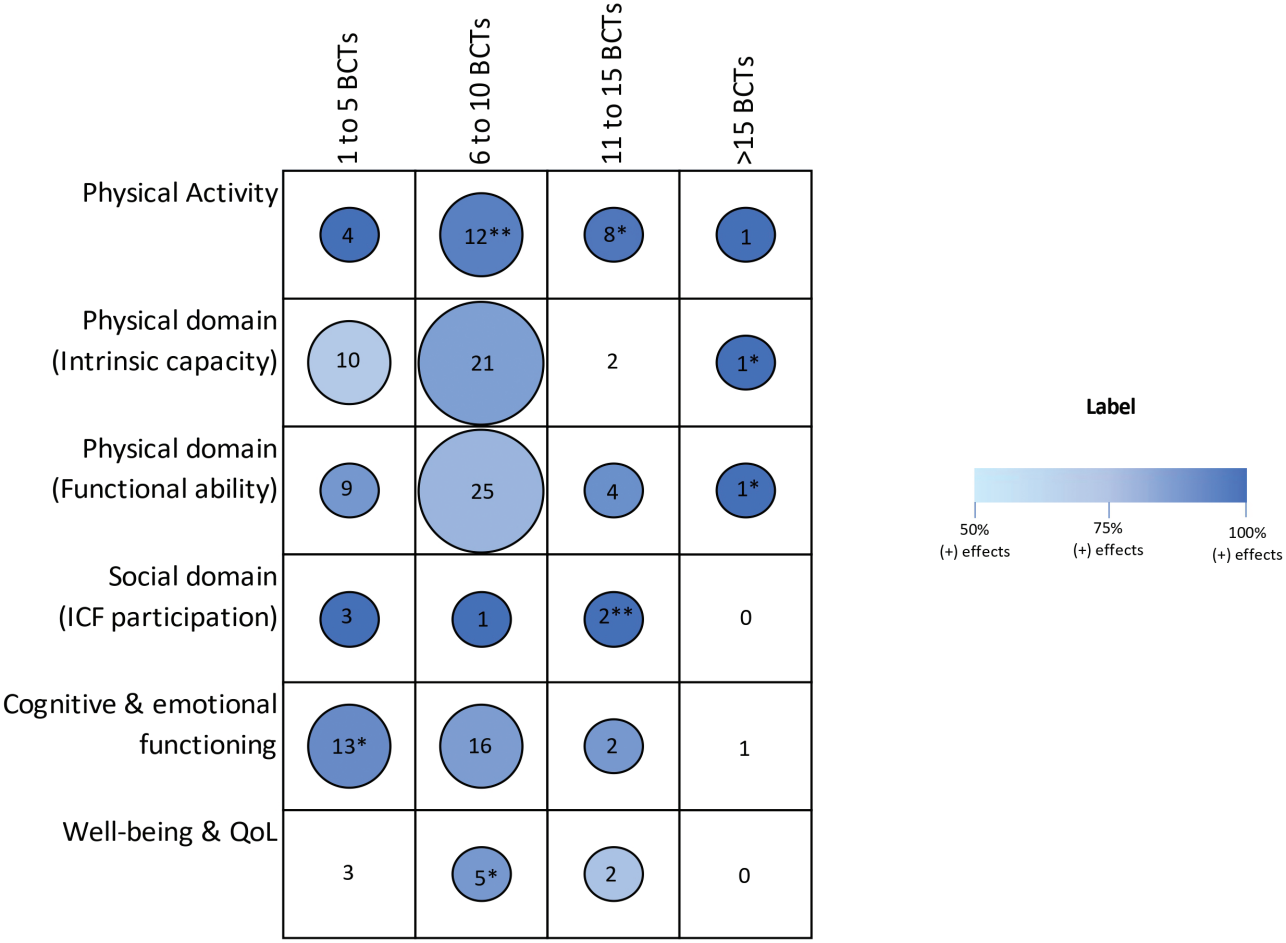
Intervention effects by number of BCTs used and outcome domain. The figure under Label presents the effect direction and statistical significance for outcome domains across primary studies. The numbers in each cell indicate the number of intervention groups. All intervention arms from the same study were included as a unique intervention group. The size of the circle depicts the number of intervention groups such that the larger the circle, the greater the number of intervention groups. Large circles represent ≥20 intervention groups; medium circles represent 10–20 intervention groups; and small circles represent <10 intervention groups. Shades of color depict the overall positive effect direction such that the darker the color, the higher the proportion of positive effects across comparisons. No shading reflects <50% positive effect or negative effects (there are none in this figure). *If 50 to 75% of total outcomes were reported as positive and statistically significant. For example, for the goals and physical activity outcomes circle, * indicates that 6–9 of 12 of the interventions tested which included the BCT goal setting had a positive and significant effect on physical activity outcome compared to their comparison no intervention groups. **If >75% of total outcomes were reported as positive and statistically significant.

Interventions targeting physical activity and social domain outcomes were highly positive, with 94%–100% positive and greater than 75% positive and significant effects for physical activity interventions compared with no interventions and 100% of overall positive effects for social domain regardless of whether 1 to 5 BCTs, 6 to 10, 11 to 15, or >15 BCTs were used (Fig. 3). For 2 of 2 physical activity interventions with 11–15 BCTs compared with no intervention and targeting the social domain, both had a positive and significant outcome.

Interventions that used 6 to 10 BCTs were associated with the most positive overall effects across all domain outcomes (72%–100% of overall positive effects and positive and significant results for more than 75% of comparisons) and studies with 11–15 BCTs had slightly more overall positive effects across all six outcome domains (33%–100%) than 1–5 BCTs (also 33%–100%). Only one study had more than 15 BCTs.

## Discussion

This review found that the physical activity programs and services for older people tested in good quality trials used eight BCTs on average, although this varied depending on type and location of intervention delivered or promoted, and the outcome of interest. Only 39 of a possible 93 discrete BCTs defined in the BCT taxonomy were used, with 10 of the BCTs accounting for almost three quarters (73%) of all the BCTs used. The most common BCTs were “action planning,” “instructions on how to perform a behavior,” and “graded tasks.” For physical activity outcomes, interventions that included the top 10 BCTs, regardless of which BCTs or groupings, had an overwhelmingly positive effect (>90% of overall positive effect), suggesting that interventions including these BCTs are appropriate for improving physical activity, and any variation in physical activity outcomes found in such studies may be related to context and content rather than BCT use. A similar pattern was seen for outcomes in the social domain. The top 10 BCTs were not, however, necessarily those identified in interventions with the most positive outcomes across other domains and there was more variation in effects found in other domains. Programs and services that used more BCTs did not necessarily have more positive effects.

Other researchers have similarly found only a small number of potential BCTs are used regularly, for example, physiotherapists use only a small number of BCTs to promote physical activity, both clinically and experimentally [40, 41]. This was attributed to factors such as lack of knowledge about BCTs and the varying presentation of the patient. However, unlike our study, the most frequently used BCT in these studies was “social support (unspecified),” focusing more on enabling the patient rather than simply instructing them. This BCT featured in less than half (30/70) of the interventions in our review, even though there is evidence to suggest it is linked with greater positive effects [21, 42]. The interventions in our review which included “social support (unspecified)” had 50%–75% positive and significant effects on outcomes of physical activity, social domain, well-being, and QoL. It may be that these outcomes are more commonly goals of physiotherapy outpatient treatment, but these findings suggest that they would be a valuable addition to other interventions that promote physical activity. Other BCTs less frequently identified in our review such as “goal setting” (16/70), and “self-monitoring” (22/70) are associated with greater intervention effects in other physical activity interventions [16, 21, 31, 39, 42, 43], again indicating that researchers may not be considering the evidence when selecting BCTs to promote behavior change in physical activity.

The potential of these BCTs is also supported by our review when we consider the trial-based between-group effects in interventions across all outcomes. Interventions that use either “goal setting,” “problem solving,” or “self-monitoring of behavior” have 72% or more overall positive effects on all outcome measures, and “social support (unspecified)” has more than 50% overall positive effects on all outcome measures. BCT groupings found in interventions with higher positive effects were “goals and planning” (however, the majority of these were “action planning” not “goal setting”), “feedback and monitoring,” “social support,” and “repetition and substitution,” having 67% or greater overall positive effects. There is one systematic review of physical activity interventions for older people which found setting behavioral goals together with prompting self-monitoring of behavior was associated with lower levels of physical activity [22]. However, our findings did not support this, nor did a systematic review investigating people living with dementia, suggesting further research is needed to explore the nuances of goal setting and self-monitoring in an older population, as well as the combinations of BCTs that may increase effectiveness, as this was not considered here beyond the BCTT groupings.

Our research adds to this body of knowledge by demonstrating that certain individual BCTs and BCT groupings, namely “goal setting,” “problem solving,” “self-monitoring of behavior,” and “social support,” are found in physical activity interventions for older people with more overall positive effects than other BCTs, across all six outcome domains, not just physical activity. The BCTs or BCT groups described in our study, however, are not necessarily the most used BCTs as described above, suggesting that, for older people at least, researchers and practitioners should be looking to the current evidence base on BCTs to develop physical activity interventions and services.

There is also a likelihood that the BCTs identified within research papers and protocols may not reflect what is actually delivered in the interventions, as a lack of treatment fidelity is a common issue for behavior change studies [44]. Process evaluation of program implementation would allow us to confirm the real-world use and application of BCTs.

With regard to the impact of other BCTs and BCT groupings, the variation between outcome measures is interesting—for example, the BCTs “social support, unspecified,” “self-monitoring of behavior,” and “information about health consequences” were the only BCTs found in interventions with more than 75% positive effects for well-being and QoL, but only “graded tasks” and “goal setting” were found in interventions with more than 75% positive effects for intrinsic capacity outcomes. The most promising BCT grouping for intrinsic capacity was “antecedents” (adding objects to the environment being the only BCT used from this grouping in these interventions). The key point here is that the same BCT may impact on different outcomes differently and BCTs may not all have the same function, therefore interventions need to be planned based on the physical activity intervention to be delivered or promoted, main outcomes of interest and possibly also location or context.

When considering the impact of the number of BCTs used on the overall effects for each domain, it is apparent that more is not necessarily better, with the most positive effects on average across all outcomes occurring in interventions with between 6 and 10 BCTs. These findings receive mixed support from other research, with some interventions showing increased effect with a greater number of BCTs [41, 45] while others found there was no difference [16, 46]. What is likely is that other factors such as content and setting of the program, as well as the function of the BCT, may have more influence than simply the number used.

While on average, these interventions used eight BCTs, this number varied significantly depending on the type of intervention. Interventions that promoted overall physical activity had more BCTs on average than those that involved structured exercise or recreation, possibly because they rely solely on interpersonal and motivational skills in, the absence of a particular program design or environment. Structured exercise is an activity, which does not necessarily require additional aspects, BCTs to be added. Similarly, interventions that took place in the participants’ own homes had more BCTs than those conducted in a community center or elsewhere, again because the goal is behavior change. Furthermore, people exercising in their own homes may be seen to need more support for behavior change, while recreation venues or residential aged care facilities can use attributes of their environments to support the program.

Physical activity interventions tested in trials that had physical activity as an outcome also used more BCTs than those that did not have physical activity as an outcome. This may be because more of an effort was made to target physical activity behavior change when it was being measured as an outcome, as opposed to, say a physical activity program with cognitive outcomes as a primary measure.

### Considerations

A key difficulty with any study of BCT use in programs and services tested in trials is the accurate reporting of BCT use in publications. To ameliorate this, we reviewed each intervention protocol, where it was available, and any other relevant publications referred to by the study, however, study protocols were not available for every study, which in itself could lead to under-reporting of BCTs in these papers. Additionally, the reporting of interventions in some publications is less than ideal and emphasizes the ongoing need to use a systematic method of reporting, such as the BCT taxonomy. Classification of the BCTs by the author also has the potential for error, although training and the initial double coding and use of a reliable, validated taxonomy goes some way to improving this.

The rapid review process undertaken for the WHO report from where we extracted our data has been detailed elsewhere [36]. The report included only good quality RCTs with at least 100 participants, which minimizes the bias in our review. Our study also adopted the same method of reporting outcomes, that is, we used a form of “vote-counting” to report the proportion of outcomes for which comparisons between groups were positive, rather than undertaking a meta-analysis to synthesize results. This method allowed for the synthesis of a much larger number of outcomes and is considered “acceptable” by the Cochrane Handbook [37] but we acknowledge that the approach we have taken does not consider the size of effects and remains inferior to meta-analysis.

A key strength of our study is its focus on between-group differences identified in randomized trials. However, this study is essentially observational as it is not possible to directly compare effects in studies including different BCTs or groups of BCTs. Our findings can provide a basis for an intervention to yield an effect similar to what has been achieved in an RCT by applying the same BCT or group of BCTs, but there is the risk of including BCTs that do not add to effectiveness but just happen to be included in effective interventions. A recent scoping review evaluating BCT efficacy methods [47] identified similar limitations in other methods. Additionally, many other factors play a part in influencing intervention outcomes, and some of the positive effects seen in our study will be attributable to other intervention components such as dose, intensity, tailoring, and duration of the intervention. Future studies should clearly specify a priori BCTs included in the intervention to be tested, including a proposed mechanism of change for a specific outcome (using logic model) so that mechanisms of effect can be explored with mediation analysis. The recently proposed Behaviour Change Technique Ontology (BCTO) [48], designed to reliably capture all aspects of the content of behavior change interventions (including mechanism of action), can be used as a way of trying to further tease out the successful components of a program.

### Implications for policy and practice

This is the first review to examine the use of BCTs in large, good quality trials testing physical activity programs and services for older people. It considers the impact that the inclusion of BCTs in interventions has on outcomes across multiple domains, including physical activity.

Interventions that improved physical activity outcomes used a range of BCTs. However, this is not the case with other outcome domains, and so we would encourage practitioners to consider the potential of different BCTs to increase the effectiveness of their physical activity interventions for different outcomes based on our findings. In particular, the use of BCTs “goal setting,” “problem solving,” and “self-monitoring of behavior” and BCT groupings “goals and planning,” “feedback and monitoring,” “social support,” and “repetition and substitution” in interventions appeared to have the most positive effects across the six domains investigated here—physical activity, intrinsic capacity (physical domain), functional ability (physical domain), social domain, cognitive and emotional functioning, and well-being and QoL. The potential relationship between different BCTs and BCT groupings and different outcomes in physical activity interventions is a key finding here for future intervention implementation. Further exploratory research could compare the effectiveness of different interventions which include other different combinations of BCTs for various outcome measures. It is also possible that some less commonly used BCTs not examined here are highly effective, and these should be considered in future research.

## Conclusion

Physical activity programs and services for older adults using BCTs improved physical activity outcomes, and different BCTs were included in programs and services that impacted on other outcome domains. It is therefore important for policymakers and program providers to consider the desired outcome/s when planning a physical activity program/service. A clear description of which BCTs are included in interventions, the proposed mechanism of effect, and evaluating their impact on different trial outcomes will help guide evidence-based practice and improve the effectiveness of future interventions. It is essential that BCTs are reported accurately, so we encourage providers to design, implement, and evaluate interventions using a BCT taxonomy, which will further strengthen the evidence base.

## Supplementary Material

kaad074_suppl_Supplementary_Material

## References

[1] Lee IM , Shiroma EJ, Lobelo F, Puska P, Blair SN, Katzmarzyk PT; Lancet Physical Activity Series Working Group. Effect of physical inactivity on major non-communicable diseases worldwide: an analysis of burden of disease and life expectancy. Lancet. 2012; 380(9838):219–229. 22818936 10.1016/S0140-6736(12)61031-9 PMC3645500

[2] WHO. WHO Guidelines on Physical Activity and Sedentary Behaviour. World Health Organization; 2020.

[3] The Physical Activity Guidelines Advisory Committee. Physical Activity Guidelines Advisory Committee Report, 2008. To the Secretary of Health and Human Services. Part A: executive summary. Nutr Rev. 2009; 67(2):114–120. 19178654 10.1111/j.1753-4887.2008.00136.x

[4] Livingston G , Huntley J, Sommerlad A, et al. Dementia prevention, intervention, and care: 2020 report of the Lancet Commission. Lancet. 2020; 396(10248):413–446. 32738937 10.1016/S0140-6736(20)30367-6 PMC7392084

[5] Guthold R , Stevens GA, Riley LM, Bull FC. Worldwide trends in insufficient physical activity from 2001 to 2016: a pooled analysis of 358 population-based surveys with 19 million participants. Lancet Glob Health. 2018; 6(10):e1077–e1086. 30193830 10.1016/S2214-109X(18)30357-7

[6] Bauman A , Merom D, Bull FC, Buchner DM, Fiatarone Singh MA. Updating the evidence for physical activity: summative reviews of the epidemiological evidence, prevalence, and interventions to promote “Active Aging”. Gerontologist. 2016; 56(Suppl_2):S268–S280. 26994266 10.1093/geront/gnw031

[7] WHO. Global action plan on physical activity 2018–2030: more active people for a healthier world. Geneva: World Health Organisation, 2018.

[8] WHO. ACTIVE: A Technical Package for Increasing PhysicalActivity. Geneva: World Health Organization, 2018.

[9] Taylor J , Walsh S, Kwok W, et al. A scoping review of physical activity interventions for older adults. Int J Behav Nutr Phys Act. 2021; 18(1):82. 34193157 10.1186/s12966-021-01140-9 PMC8243293

[10] Baldwin JN , Hassett L, Sherrington C. Framework to classify physical activity intervention studies for older adults. Transl J Am Coll Sports Med. 2023; 8(3):e000230.

[11] Zhou Y , Ma L. Intrinsic capacity in older adults: recent advances. Aging Dis. 2022; 13(2):353–359. 35371613 10.14336/AD.2021.0818 PMC8947834

[12] WHO. World report on ageing and health. 2015. Available at https://www.who.int/publications/i/item/9789241565042. Accessibility verified July 10, 2023.

[13] WHO. International classification of functioning, disability, and health: children & youth version: ICF-CY. Geneva: World Health Organization (WHO), 2007.

[14] Cashin AG , McAuley JH. Clinimetrics: Physiotherapy Evidence Database (PEDro) scale. J Physiother. 2020; 66(1):59. 31521549 10.1016/j.jphys.2019.08.005

[15] Pinheiro MB , Oliveira JS, Baldwin JN, et al. Impact of physical activity programs and services for older adults: a rapid review. *Int J Behav Nutr Phys Act.* 2022; 19:87. 35836187 10.1186/s12966-022-01318-9 PMC9284866

[16] Michie S , Abraham C, Whittington C, McAteer J, Gupta S. Effective techniques in healthy eating and physical activity interventions: a meta-regression. Health Psychol. 2009; 28(6):690–701. 19916637 10.1037/a0016136

[17] Michie S , Abraham C, Eccles MP, Francis JJ, Hardeman W, Johnston M. Strengthening evaluation and implementation by specifying components of behaviour change interventions: a study protocol. Implement Sci. 2011; 6(1):10–10. 21299860 10.1186/1748-5908-6-10 PMC3041694

[18] Patterson K , Davey R, Keegan R, Kunstler B, Woodward A, Freene N. Behaviour change techniques in cardiovascular disease smartphone apps to improve physical activity and sedentary behaviour: systematic review and meta-regression. Int J Behav Nutr Phys Act. 2022; 19(1):81. 35799263 10.1186/s12966-022-01319-8 PMC9261070

[19] Howlett N , Trivedi D, Troop NA, Chater AM. Are physical activity interventions for healthy inactive adults effective in promoting behavior change and maintenance, and which behavior change techniques are effective? A systematic review and meta-analysis. Transl Behav Med. 2019; 9(1):147–157. 29506209 10.1093/tbm/iby010 PMC6305562

[20] Kunstler BE , Cook JL, Kemp JL, O’Halloran PD, Finch CF. The behaviour change techniques used by Australian physiotherapists to promote non-treatment physical activity to patients with musculoskeletal conditions. J Sci Med Sport. 2019; 22(1):2–10. 30554613 10.1016/j.jsams.2018.06.002

[21] Nyman SR , Adamczewska N, Howlett N. Systematic review of behaviour change techniques to promote participation in physical activity among people with dementia. Br J Health Psychol. 2018; 23(1):148–170. 28980370 10.1111/bjhp.12279

[22] French DP , Olander EK, Chisholm A, Mc Sharry J. Which behaviour change techniques are most effective at increasing older adults’ self-efficacy and physical activity behaviour? A systematic review. Ann Behav Med. 2014; 48(2):225–234. 24648017 10.1007/s12160-014-9593-z

[23] Samdal GB , Eide GE, Barth T, Williams G, Meland E. Effective behaviour change techniques for physical activity and healthy eating in overweight and obese adults; systematic review and meta-regression analyses. Int J Behav Nutr Phys Act. 2017; 14(1):42. 28351367 10.1186/s12966-017-0494-y PMC5370453

[24] Eisele A , Schagg D, Kramer LV, Bengel J, Gohner W. Behaviour change techniques applied in interventions to enhance physical activity adherence in patients with chronic musculoskeletal conditions: a systematic review and meta-analysis. Patient Educ Couns. 2019; 102(1):25–36. 30279029 10.1016/j.pec.2018.09.018

[25] Hailey V , Rojas-Garcia A, Kassianos AP. A systematic review of behaviour change techniques used in interventions to increase physical activity among breast cancer survivors. Breast Cancer. 2022; 29(2):193–208. 34989962 10.1007/s12282-021-01323-z PMC8885559

[26] Grimmett C , Corbett T, Brunet J, et al. Systematic review and meta-analysis of maintenance of physical activity behaviour change in cancer survivors. Int J Behav Nutr Phys Act. 2019; 16(1):37–37. 31029140 10.1186/s12966-019-0787-4 PMC6486962

[27] Hallward L , Patel N, Duncan LR. Behaviour change techniques in physical activity interventions for men with prostate cancer: a systematic review. Systematic review. J Health Psychol. 2020; 25(1):105–122. 29446325 10.1177/1359105318756501

[28] Nyman SR. Which behaviour change techniques are effective in promoting physical activity among older people with dementia? A call for research into three underexplored avenues. J Aging Phys Act. 2019; 27(4):441–445. 30676205 10.1123/japa.2018-0301

[29] Beard JR , Officer A, De Carvalho IA, et al. The world report on ageing and health: a policy framework for healthy ageing. Lancet. 2016; 387(10033):2145–2154. 26520231 10.1016/S0140-6736(15)00516-4 PMC4848186

[30] van Stralen MM , De Vries H, Mudde AN, Bolman C, Lechner L. Determinants of initiation and maintenance of physical activity among older adults: a literature review. Health Psychol Rev. 2009; 3(2):147–207.

[31] Room J , Hannink E, Dawes H, Barker K. What interventions are used to improve exercise adherence in older people and what behavioural techniques are they based on? A systematic review. BMJ Open. 2017; 7(12):e019221–e019221. 10.1136/bmjopen-2017-019221 PMC5736048 29247111

[32] Michie S , Richardson M, Johnston M, et al. The behavior change technique taxonomy (v1) of 93 hierarchically clustered techniques: building an international consensus for the reporting of behavior change interventions. Ann Behav Med. 2013; 46(1):81–95. 23512568 10.1007/s12160-013-9486-6

[33] Dusseldorp E , van Genugten L, van Buuren S, Verheijden MW, van Empelen P. Combinations of techniques that effectively change health behavior: evidence from Meta-CART analysis. Health Psychol. 2014; 33(12):1530–1540. 24274802 10.1037/hea0000018

[34] O’Brien N , McDonald S, Araújo-Soares V, et al. The features of interventions associated with long-term effectiveness of physical activity interventions in adults aged 55-70 years: a systematic review and meta-analysis. Health Psychol Rev. 2015; 9(4):417–433. 25689096 10.1080/17437199.2015.1012177

[35] Tang MY , Smith DM, Mc Sharry J, Hann M, French DP. Behavior change techniques associated with changes in postintervention and maintained changes in self-efficacy for physical activity: a systematic review with meta-analysis. Ann Behav Med. 2018; 53(9):801–815. 10.1093/abm/kay090 30534971

[36] Pinheiro MB , Oliveira JS, Baldwin JN, et al. Impact of physical activity programs and services for older adults: a rapid review. Int J Behav Nutr Phys Act. 2022; 19(1):87. 35836187 10.1186/s12966-022-01318-9 PMC9284866

[37] McKenzie JE , Brennan SE. Synthesizing and Presenting Findings Using Other Methods. Chichester, UK: John Wiley & Sons, Ltd.; 2019:321–347.

[38] Kwok WS , Sherrington C, Nyman SR, Oliveira J, Tiedemann A, Pinheiro M. Use of behaviour change techniques (BCTs) in exercise interventions targeting falls prevention and adherence to supervised exercise in community-dwelling older adults: protocol for a systematic review. Available at https://osf.io/jfmw/ 10.1123/jpah.2024-0830 40973057

[39] McEwan D , Harden SM, Zumbo BD, et al. The effectiveness of multi-component goal setting interventions for changing physical activity behaviour: a systematic review and meta-analysis. Health Psychol Rev. 2016; 10(1):67–88. 26445201 10.1080/17437199.2015.1104258

[40] Keogh A , Tully MA, Matthews J, Hurley DA. A review of behaviour change theories and techniques used in group based self-management programmes for chronic low back pain and arthritis. Man Ther. 2015; 20(6):727–735. 25865062 10.1016/j.math.2015.03.014

[41] Kunstler BE , Cook JL, Freene N, et al. Physiotherapists use a small number of behaviour change techniques when promoting physical activity: a systematic review comparing experimental and observational studies. J Sci Med Sport. 2018; 21(6):609–615. 29233466 10.1016/j.jsams.2017.10.027

[42] Willett M , Duda J, Fenton S, Gautrey C, Greig C, Rushton A. Effectiveness of behaviour change techniques in physiotherapy interventions to promote physical activity adherence in lower limb osteoarthritis patients: a systematic review. PLoS One. 2019; 14(7):e0219482. 31291326 10.1371/journal.pone.0219482 PMC6619772

[43] Schroe H , Van Dyck D, De Paepe A, et al. Which behaviour change techniques are effective to promote physical activity and reduce sedentary behaviour in adults: a factorial randomized trial of an e- and m-health intervention. Int J Behav Nutr Phys Act. 2020; 17(1):127. 33028335 10.1186/s12966-020-01001-x PMC7539442

[44] Bellg AJ , Borrelli B, Resnick B, et al; Treatment Fidelity Workgroup of the NIH Behavior Change Consortium. Enhancing treatment fidelity in health behavior change studies: best practices and recommendations from the NIH Behavior Change Consortium. Health Psychol. 2004; 23(5):443–451. 15367063 10.1037/0278-6133.23.5.443

[45] Bishop FL , Fenge-Davies AL, Kirby S, Geraghty AWA. Context effects and behaviour change techniques in randomised trials: a systematic review using the example of trials to increase adherence to physical activity in musculoskeletal pain. Psychol Health. 2015; 30(1):104–121. 25109300 10.1080/08870446.2014.953529

[46] Dombrowski SU , Sniehotta FF, Avenell A, Johnston M, MacLennan G, Araújo-Soares V. Identifying active ingredients in complex behavioural interventions for obese adults with obesity-related co-morbidities or additional risk factors for co-morbidities: a systematic review. Health Psychol Rev. 2012; 6(1):7–32.

[47] Michie S , West R, Sheals K, Godinho CA. Evaluating the effectiveness of behavior change techniques in health-related behavior: a scoping review of methods used. Transl Behav Med. 2018; 8(2):212–224. 29381786 10.1093/tbm/ibx019 PMC6062857

[48] Marques M , Wright A, Corker E, et al. The behaviour change technique ontology: transforming the behaviour change technique taxonomy v1 [version 1; peer review: 4 approved]. Wellcome Open Res. 2023; 8(308) accessible at: 10.12688/wellcomeopenres.19363.1 PMC10427801 37593567

