# Supplementary material for: Use of behavior change techniques in physical activity programs and services for older adults: findings from a rapid review
Source: Ann Behav Med. 2024 Feb 1;58(3):216–26. doi: 10.1093/abm/kaad074 (PMC10858305; doi:10.1093/abm/kaad074)
Supplement: kaad074_suppl_Supplementary_Material [file kaad074_suppl_supplementary_material.docx]

# APPENDIX 1.

## Eligibility criteria and search strategies used to identify systematic reviews (1)

Search strategies were designed by the lead author of the rapid review, with support from experienced librarians and in consultation with the senior researchers who were experienced in systematic reviews.

### Eligibility criteria for systematic reviews

| **Category** | **Inclusion** | **Exclusion** |
| --- | --- | --- |
| Publication language | Full text in English | Abstract-only, other languages |
| Publication date | Published after 1 January 2010 | Published before 1 January 2010 |
| Publication status | Published in peer-reviewed journals | Unpublished reviews |
| Study design | - Systematic review - Meta-analysis | - Narrative/literature review - Commentaries - Editorials - Protocols - Controlled, pre-post trials - Cohort/cross-sectional studies - Reviews of reviews (meta-reviews) - Scoping reviews |
| Characteristics of individual studies | | |
| Population | - Adults 60+ years: healthy; frail; or with a disability. - Reviews including younger participants where average age >=60 years - Bone mineral density conditions - Mild cognitive impairment - Sarcopenia - Obesity - Mixed chronic conditions | - Samples selected for pre-existing medical conditions (e.g. stroke, dementia, Parkinson’s) - Reviews including younger participants where the average age is <60 years |
| Intervention | Question 1 (All PA interventions):   - Physical activity - Complex and/or multifaceted interventions - Health professional education - Community-wide campaigns - Age-friendly environments - Policy   Question 2 (PA programmes):   - Supervised/unsupervised - All physical activity types - All PA providers - All programme delivery modes | Question 1:  No exclusions where the primary focus is increasing physical activity. Question 2:   - Programmes without participant-based PA outcomes. - Perturbation/platform training - Combined interventions (e.g. supplement/nutrition and PA) - Occupational therapy/ physiotherapy without PA |
| Comparators | All included | NA |
| Outcomes | Question 1: Physical activity  Question 2: Longitudinal physical activity, disability, falls, mood, health-related quality of life.  Cognitive function  Strength and fitness  Balance  Laboratory measures of cognitive function  Any outcomes that measure physical activity, participation/social, physical function, cognitive and emotional, global measures (e.g. quality of life, frailty, functioning, wellbeing) | - Disease- or disorder-specific symptoms or laboratory measures. e.g. blood glucose; cholesterol. - Programmes without longitudinal outcomes - Adherence-only - Barriers & facilitators only |
| Settings | Recruitment in all settings, but programmes delivered in:   - Healthcare settings - Residential care - Community/home settings | Programmes delivered in inpatient settings |

### Search strategies

#### MEDLINE search strategy

| **Description** | **Search terms** |
| --- | --- |
| Limit: language and exclude animal only | (English[lang]) NOT ("Animals"[Mesh] NOT ("Animals"[Mesh] AND "Humans"[Mesh])) |
| Population | AND "old"[tiab] OR "older"[tiab] OR "aged"[tiab] OR "aging"[tiab] OR "ageing"[tiab] OR "elderly” [tiab] |
| Limit: age groups | NOT (("infant"[Mesh] OR "child"[mesh] OR "adolescent"[mh])  NOT (("infant"[Mesh] OR "child"[mesh] OR "adolescent"[mh]) AND "adult"[Mesh])) |
| Limit: date | AND ("2010/01/01"[PDAT]: "3000/12/31"[PDAT]) |
| Publication type | AND (systematic[sb] OR meta-analysis[pt] OR “systematic review” [tiab] OR “systematic literature review” [tiab] OR metaanalysis[tiab] OR "meta analysis"[tiab] OR metanalyses[tiab] OR "meta analyses"[tiab] OR "pooled analysis"[tiab] OR “pooled analyses” [tiab] OR "pooled data"[tiab]) |
| Limit: publication type | NOT (“comment” [Publication Type] OR “editorial” [Publication Type]) |
| Physical activity: | AND (("Exercise"[mh] OR "Exercise"[tiab] OR "Physical activity"[tiab] OR "Lifestyle activities"[tiab] OR "Lifestyle activity"[tiab] OR "Recreational activities"[tiab] OR "Recreational activity"[tiab] OR "Tai ji"[mh] OR "Yoga"[mh] OR "Activities of daily living"[tiab] OR "Activity of daily living"[tiab] OR "Free living activities"[tiab] OR "Free living activity"[tiab] OR "Balance training"[tiab] OR "Qigong"[mh] OR "Functional training"[tiab]) OR (("Aerobic activities"[tiab] OR "Aerobic activity"[tiab] OR "Cardiovascular activities"[tiab] OR "Cardiovascular activity"[tiab] OR "Endurance activities"[tiab] OR "Endurance activity"[tiab] OR "Physical activities"[tiab] OR "Physical conditioning"[tiab] OR "Resistance training"[tiab] OR "strength training"[tiab] OR "Tai chi"[tiab] OR "Tai ji"[tiab] OR "Yoga"[tiab] OR "Walk"[tiab] OR "Walking"[tiab] OR "Chi kung"[tiab] OR "Qigong"[tiab] OR "stretching"[tiab]) NOT medline[sb])) |

#### CINAHL search strategy

| **Description** | **Search Terms** |
| --- | --- |
| Physical activity | 1. (MH "Exercise+") 2. (TI exercise OR AB exercise) 3. (TI "physical activity" OR AB "physical activity") 4. TI "Lifestyle activities" OR AB "Lifestyle activities" OR TI "Lifestyle activity" OR AB "Lifestyle activity" 5. TI "Recreational activities" OR AB "Recreational activities" OR TI "Recreational activity" OR AB "Recreational activity" 6. (MH "Tai Chi") 7. (MH "Yoga+") 8. (MH "Activities of Daily Living+") 9. TI "Activities of daily living" OR AB "Activities of daily living" 10. TI "Free living activity" OR AB "Free living activity" 11. TI "Balance training" OR AB "Balance training" 12. (MH "Qigong") 13. TI "Functional training" OR AB "Functional training" 14. TI "Aerobic activities" OR AB "Aerobic activities" OR TI "Aerobic activity" OR AB "Aerobic activity" 15. TI "Cardiovascular activities" OR AB "Cardiovascular activities" OR TI "Cardiovascular activity" OR AB "Cardiovascular activity" 16. TI "Endurance activities" OR AB "Endurance activities" OR TI "Endurance activity" OR AB "Endurance activity" 17. TI "Physical activities" OR AB "Physical activities" 18. TI "Physical conditioning" OR AB "Physical conditioning" 19. TI "Resistance training" OR AB "Resistance training" 20. TI "strength training" OR AB "strength training" 21. TI "Tai chi" OR AB "Tai chi" OR TI "Tai ji" OR AB "Tai ji" 22. TI Yoga OR AB Yoga 23. TI Walk OR AB Walk OR TI walking OR AB walking 24. TI "Chi kung" OR AB "Chi kung" OR TI "Qigong" OR AB "Qigong" 25. TI stretching OR AB stretching |
| Combine physical activity | 1. 1-25 OR |
| Limit publication type | 1. (MH "Systematic Review") 2. TI systematic review OR AB systematic review 3. TI systematic literature review OR AB systematic literature review 4. TI metaanalysis OR AB metaanalysis OR TI meta analysis OR AB meta analysis 5. TI metaanalyses OR AB metaanalyses OR TI meta analyses OR AB meta analyses 6. TI meta-analysis OR AB meta-analysis OR TI meta-analyses OR AB meta-analyses 7. TI "pooled analysis" OR AB "pooled analysis" OR TI "pooled analyses" OR AB "pooled analyses" 8. TI "pooled data" OR AB "pooled data" |
| Combine publication type | 1. 27-34 OR |
| Limit age group | 1. TI "old" OR TI "older" OR TI "aged” OR TI "aging" OR TI "ageing" OR TI "elderly” OR AB "old" OR AB "older" OR AB "aged” OR AB "aging" OR AB "ageing" OR AB "elderly” |
| Combine all | 1. 26 AND 35 AND 36 |
| Limits | 1. Limit to English AND Date>01/01/2010 AND exclude MEDLINE |

#### PEDro search strategy

| **Abstract & Title:** | Physical activity | Exercise |  |  |  |  |
| --- | --- | --- | --- | --- | --- | --- |
| **Therapy:** |  |  | Fitness training | Health promotion | Skill training | Strength training |
| **Subdiscipline:** | Gerontology | Gerontology | Gerontology | Gerontology | Gerontology | Gerontology |
| **Method:** | Systematic review | Systematic review | Systematic review | Systematic review | Systematic review | Systematic review |
| **Published since:** | 2010 | 2010 | 2010 | 2010 | 2010 | 2010 |

#### Cochrane search strategy

| **Description** | **Search terms** |
| --- | --- |
| Physical activity | 1. MeSH descriptor: [Exercise] explode all trees 2. (exercise): ti, ab, kw 3. (physical activity): ti, ab, kw 4. (Lifestyle activities): ti, ab, kw OR (Lifestyle activity): ti, ab, kw 5. (Recreational activities): ti, ab, kw OR (Recreational activity): ti, ab, kw 6. MeSH descriptor: [Tai Ji] explode all trees 7. MeSH descriptor: [Yoga] explode all trees 8. (Activities of Daily Living): ti, ab, kw 9. MeSH descriptor: [Activities of Daily Living] explode all trees 10. (Free living activity): ti, ab, kw 11. (Balance training): ti, ab, kw 12. MeSH descriptor: [Qigong] explode all trees 13. (Functional training): ti, ab, kw 14. (Aerobic activities): ti, ab, kw OR (Aerobic activity): ti, ab, kw 15. (Cardiovascular activities): ti, ab, kw OR (Cardiovascular activity): ti, ab, kw 16. (Endurance activities): ti, ab, kw OR (Endurance activity): ti, ab, kw 17. (Physical activities): ti, ab, kw 18. (Physical conditioning): ti, ab, kw 19. (Resistance training): ti, ab, kw 20. (strength training): ti, ab, kw 21. (Tai chi): ti, ab, kw OR (Tai Ji): ti, ab, kw 22. (Yoga): ti, ab, kw 23. (Walk): ti, ab, kw OR (Walking): ti, ab, kw 24. (Chi kung): ti, ab, kw OR (Qigong): ti, ab, kw 25. (stretching): ti, ab, kw |
| Combine physical activity | 1. #1 OR #2 OR #3 OR #4 OR #5 OR #6 OR #7 OR #8 OR #9 OR #10 OR #11 OR #12 OR #13 OR #14 OR #15 OR #16 OR #17 OR #18 OR #19 OR #20 OR #21 OR #22 OR #23 OR #24 OR #25 |
| Limit publication type | 1. MeSH descriptor: [Systematic Review] explode all trees 2. (Systematic Review): ti, ab, kw 3. (systematic literature review): ti, ab, kw 4. (metaanalysis): ti, ab, kw OR (meta analysis): ti, ab, kw 5. (metaanalyses): ti, ab, kw OR (meta analyses): ti, ab, kw 6. (meta-analysis): ti, ab, kw OR (meta-analyses): ti, ab, kw 7. (pooled analysis): ti, ab, kw OR (pooled analyses): ti, ab, kw 8. (pooled data): ti, ab, kw |
| Combine publication type | 1. #27 OR #28 OR #29 OR #30 OR #31 OR #32 OR #33 OR #34 |
| Limit age group | 1. (old): ti, ab, kw OR (older): ti, ab, kw OR (aged): ti, ab, kw OR (ageing): ti, ab, kw AND (aging): ti, ab, kw 2. (elderly): ti, ab, kw |
| Combine age group | 1. #26 AND #35 AND (#36 OR #37) |
| Limit date | 1. Limits: with Cochrane Library publication date from Jan 2010 to Nov 2020, in Cochrane Reviews. |

# APPENDIX 2.

## Search strategies for the selection of primary studies investigating sports for older adults (1)

Search strategies were designed by the lead author of the rapid review, with support from experienced librarians and in consultation with the senior researchers who were experienced in systematic reviews.

### Medline (Ovid)

1. Baseball/ or Basketball/ or Bicycling/ or Boxing/ or Football/ or Golf/ or Gymnastics/ or Hockey/ or exp Martial Arts/ or exp Racquet Sports/ or cricket sport/ or team sports/
2. Return to Sport/ or exp Running/ or Skating/ or exp Snow Sports/ or Soccer/ or exp Swimming/
3. Volleyball/ or exp Water Sports/ or Weight Lifting/ or Wrestling/
4. "Track and Field"/
5. (mountain bik* or sports or AFL or alpine ski* or archery or athletics or badminton or basketball or biathlon or biking or Boxing or canoe* or cricket or cross country ski* or curling).tw.
6. (cycling or diving or duathlon or equestrian or fencing or football or golf or gymnastics or Handball or hippotherapy or Hockey or horseback riding or horse riding or judo or kayak or kickboxing or lawn bowls or bowling).tw.
7. (marathon or netball or badminton or snowboard or triathlon or Polo or powerlifting or runn* or rowing or sailing or shooting or Skating or skiing or snowboard or soccer or sport*).tw.
8. (surfing or swimming or table tennis or taekwondo or Tae Kwon Do or tenpin bowling or Tennis or Trampolin* or triathlon or volleyball or volley).tw.
9. (australian football or baseball or fencing or racing or rugby or sport* or tennis or union or league).tw.
10. 1 or 2 or 3 or 4 or 5 or 6 or 7 or 8 or 9
11. *Aged/ or *"Aged, 80 and over"/
12. exp Aged/ not Adolescent.mp.
13. (elderly or seniors or geriatric).tw.
14. (older adj (adult or people or person$1)).tw.
15. 11 or 12 or 13 or 14
16. Randomized Controlled Trials as Topic/
17. Random Allocation/
18. Controlled Clinical Trials as Topic/
19. Control Groups/
20. Double-Blind Method/
21. Single-Blind Method/
22. Placebos/
23. randomized controlled trial.pt.
24. controlled clinical trial.pt.
25. (random$ or RCT or RCTs).tw.
26. (controlled adj5 (trial$ or stud$)).tw.
27. (clinical$ adj5 trial$).tw.
28. (randomi?ed adj5 trial).mp.
29. 16 or 17 or 18 or 19 or 20 or 21 or 22 or 23 or 24 or 25 or 26 or 27 or 28
30. exp Animals/ not Humans/
31. 29 not 30
32. 10 and 15 and 31

**Results: 3391 on 19 April 2021**

### CINAHL (Ebsco)

S1 (MH "Baseball" OR "Basketball" OR "Cycling" OR "Boxing" OR "Football" OR "Golf" OR "Gymnastics" OR "Hockey" OR "Martial Arts" OR "Racquet Sports+" OR "Sports Re-Entry" OR "Running+" OR "Skating+" OR "Winter Sports+" OR "Soccer" OR "Sports" OR "Swimming" OR "Track and Field" OR "Volleyball" OR "Aquatic Sports+" OR "Weight Lifting" OR "Wrestling" OR "Sports Participation" OR "Bowling" OR "Fencing" OR "Handball" OR "Race Walking" OR "Skiing+" OR "Team Sports+" OR "Triathlon")

S2 (TI "mountain bik*" OR "sports" OR AFL OR "alpine ski*" OR archery OR athletics OR badminton OR basketball OR biathlon OR biking OR Boxing OR canoe* OR cricket OR "cross#country ski*" OR curling OR cycling OR diving OR duathlon OR equestrian OR fencing OR football)

S3 (AB "mountain bik*" OR "sports" OR AFL OR "alpine ski*" OR archery OR athletics OR badminton OR basketball OR biathlon OR biking OR Boxing OR canoe* OR cricket OR "cross#country ski*" OR curling OR cycling OR diving OR duathlon OR equestrian OR fencing OR football)

S4 (TI golf OR gymnastics OR Handball OR hippotherapy OR Hockey OR "horseback riding" OR "horse riding" OR judo OR kayak OR kickboxing OR "lawn bowls" OR bowling OR marathon OR netball OR badminton OR snowboard OR Triathlon OR Polo OR powerlifting OR runn* OR rowing OR sailing)

S5 (AB golf OR gymnastics OR Handball OR hippotherapy OR Hockey OR "horseback riding" OR "horse riding" OR judo OR kayak OR kickboxing OR "lawn bowls" OR bowling OR marathon OR netball OR badminton OR snowboard OR Triathlon OR Polo OR powerlifting OR runn* OR rowing OR sailing)

S6 (TI shooting OR Skating OR skiing OR snowboard OR soccer OR sport* OR surfing OR swimming OR "table tennis" OR taekwondo OR "Tae Kwon Do" OR "tenpin bowling" OR Tennis OR Trampolin* OR triathlon OR volleyball OR volley)

S7 (AB shooting OR Skating OR skiing OR snowboard OR soccer OR sport* OR surfing OR swimming OR "table tennis" OR taekwondo OR "Tae Kwon Do" OR "tenpin bowling" OR Tennis OR Trampolin* OR triathlon OR volleyball OR volley)

S8 (TI "australian football" OR "baseball" OR " curling" OR "fencing" OR " racing" OR "rugby" OR " sport*" OR "winter sports" OR Wrestling)

S9 (AB "australian football" OR "baseball" OR " curling" OR "fencing" OR " racing" OR "rugby" OR " sport*" OR "winter sports" OR Wrestling)

S10 S1 OR S2 OR S3 OR S4 OR S5 OR S6 OR S7 OR S8 OR S9

S11 (MH "Randomized Controlled Trials") OR (MH "Clinical Trials+") OR "Randomized Controlled Trial"

S12 (MH "Aged+") OR (MH "Aged, 80 and Over")

S13 S10 AND S11 AN S12

**Results: 1012 on 19 April 2021**

### SPORTDiscus (ebsco)

S1 DE "BASEBALL" OR DE "BASKETBALL" OR DE "CYCLING" OR DE "BOXING" OR DE "FOOTBALL" OR DE "GOLF" OR DE "GYMNASTICS" OR DE "HOCKEY" OR DE "MARTIAL Arts" OR DE "RACQUETBALL" OR DE "SPORT for all" OR DE "RUNNING" OR DE "SKATING" OR DE "WINTER sports" OR DE "SOCCER"

S2 DE " sports" OR DE "MOTORSOCCER" OR DE "RUGBALL" OR DE " bowling" OR DE " dance sport" OR DE " fencing" OR DE " hockey" OR DE "road racing" OR DE "rugby" OR DE " soccer" OR DE " sports competitions" OR DE " tennis" OR DE " track & field" OR DE " workouts"

S3 DE "SWIMMING" OR DE "VOLLEYBALL" OR DE "AQUATIC sports" OR DE "AQUATIC sports competitions" OR DE "CANOE polo" OR DE "CANOES & canoeing" OR DE "DIVING" OR DE "DRAGON boat racing" OR DE "FISHING" OR DE "KNEEBOARDING" OR DE "RAFTING (Sports)" OR DE "REGATTAS" OR DE "ROWING" OR DE "SAILBOAT racing" OR DE "SAILING" OR DE "SURFING" OR DE "WATER polo" OR DE "WATER skiing" OR DE "WHITEWATER kayaking" OR DE "WHITEWATER rafting"

S4 DE "WEIGHT lifting" OR DE "WEIGHT lifting competitions" OR DE "WRESTLING" OR DE "SPORTS participation" OR DE "BOWLING" OR DE "FENCING" OR DE "HANDBALL" OR DE "WALKING (Sports)" OR DE "SKIS & skiing" OR DE "TEAM sports" OR DE "TRIATHLON"

S5 TI (mountain bik* OR sports OR AFL OR alpine ski* OR archery OR athletics OR badminton OR basketball OR biathlon OR biking OR Boxing OR canoe* OR cricket OR cross#country ski* OR curling OR cycling OR diving OR duathlon OR equestrian OR fencing OR football)

S6 TI (golf OR gymnastics OR Handball OR hippotherapy OR Hockey OR horseback riding OR horse riding OR judo OR kayak OR kickboxing OR lawn bowls OR bowling OR marathon OR netball OR snowboard OR triathlon OR Polo OR powerlifting OR runn* OR rowing OR sailing)

S7 TI (shooting OR Skating OR skiing OR snowboard OR soccer OR sport* OR surfing OR swimming OR table tennis OR taekwondo OR Tae Kwon Do OR tenpin bowling OR Tennis OR Trampolin* OR triathlon OR volleyball OR volley)

S8 TI (aussie rules OR australian football OR baseball OR fencing OR racing OR rugby OR winter sports OR Wrestling)

S9 AB (mountain bik* OR sports OR AFL OR alpine ski* OR archery OR athletics OR badminton OR basketball OR biathlon OR biking OR Boxing OR canoe* OR cricket OR cross#country ski* OR curling OR cycling OR diving OR duathlon OR equestrian OR fencing OR football)

S10 AB (golf OR gymnastics OR Handball OR hippotherapy OR Hockey OR horseback riding OR horse riding OR judo OR kayak OR kickboxing OR lawn bowls OR bowling OR marathon OR netball OR snowboard OR triathlon OR Polo OR powerlifting OR runn* OR rowing OR sailing)

S11 AB (shooting OR Skating OR skiing OR snowboard OR soccer OR sport* OR surfing OR swimming OR table tennis OR taekwondo OR Tae Kwon Do OR tenpin bowling OR Tennis OR Trampolin* OR triathlon OR volleyball OR volley)

S12 AB (aussie rules OR australian football OR baseball OR fencing OR racing OR rugby OR winter sports OR Wrestling)

S13 S1 OR S2 OR S3 OR S4 OR S5 OR S6 OR S7 OR S8 OR S9 OR S10 OR S11 OR S12

S14 controlled trial OR clinical trial

S15 random*

S16 random sampling

S17 clinic* W5 trial*

S18 random allocation

S19 randomized OR randomised

S20 randomized controlled trials

S21 S14 OR S15 OR S16 OR S17 OR S18 OR S19 OR S20

S22 Older person OR older people OR older adult*

S23 "elderly" or "senior"

S24 DE "OLDER people" OR DE "AGING" OR DE "GERIATRICS" OR DE "RETIREMENT"

S25 S22 OR S23 OR S24

S26 S13 AND S21 AND S25

**Results: 365 on 19 April 2021**

### PEDro

Sport (AB TI) AND gerontology (Subdiscipline) AND Clinical trial (method)

**Results: 24**

Skiing (AB TI) AND gerontology (Subdiscipline) AND Clinical trial (method)

**Results: 23**

archery (AB TI) AND gerontology (Subdiscipline) AND Clinical trial (method)

**Results: 0**

athletics (AB TI) AND gerontology (Subdiscipline) AND Clinical trial (method)

**Results: 0**

badminton (AB TI) AND gerontology (Subdiscipline) AND Clinical trial (method)

**Results: 0**

basketball (AB TI) AND gerontology (Subdiscipline) AND Clinical trial (method)

**Results: 1**

biathlon* (AB TI) AND gerontology (Subdiscipline) AND Clinical trial (method)

**Results: 0**

biking (AB TI) AND gerontology (Subdiscipline) AND Clinical trial (method)

**Results: 0**

cycling (AB TI) AND gerontology (Subdiscipline) AND Clinical trial (method)

**Results: 54**

boxing (AB TI) AND gerontology (Subdiscipline) AND Clinical trial (method)

**Results: 7**

canoe* (AB TI) AND gerontology (Subdiscipline) AND Clinical trial (method)

**Results: 1**

cricket (AB TI) AND gerontology (Subdiscipline) AND Clinical trial (method)

**Results: 0**

curling (AB TI) AND gerontology (Subdiscipline) AND Clinical trial (method)

**Results: 1**

Diving (AB TI) AND gerontology (Subdiscipline) AND Clinical trial (method)

**Results: 0**

duathlon* (AB TI) AND gerontology (Subdiscipline) AND Clinical trial (method)

**Results: 0**

equestrian (AB TI) AND gerontology (Subdiscipline) AND Clinical trial (method)

**Results: 0**

fencing (AB TI) AND gerontology (Subdiscipline) AND Clinical trial (method)

**Results: 0**

football (AB TI) AND gerontology (Subdiscipline) AND Clinical trial (method)

**Results: 3**

golf (AB TI) AND gerontology (Subdiscipline) AND Clinical trial (method)

**Results: 3**

gymnastics (AB TI) AND gerontology (Subdiscipline) AND Clinical trial (method)

**Results: 16**

handball (AB TI) AND gerontology (Subdiscipline) AND Clinical trial (method)

**Results: 1**

hockey (AB TI) AND gerontology (Subdiscipline) AND Clinical trial (method)

**Results: 0**

horseback riding (AB TI) AND gerontology (Subdiscipline) AND Clinical trial (method)

**Results: 1**

horse riding (AB TI) AND gerontology (Subdiscipline) AND Clinical trial (method)

**Results: 3**

judo (AB TI) AND gerontology (Subdiscipline) AND Clinical trial (method)

**Results: 0**

kayak (AB TI) AND gerontology (Subdiscipline) AND Clinical trial (method)

**Results: 3**

kickboxing (AB TI) AND gerontology (Subdiscipline) AND Clinical trial (method)

**Results: 0**

lawn bowls (AB TI) AND gerontology (Subdiscipline) AND Clinical trial (method)

**Results: 0**

marathon (AB TI) AND gerontology (Subdiscipline) AND Clinical trial (method)

**Results:0**

netball (AB TI) AND gerontology (Subdiscipline) AND Clinical trial (method)

**Results:0**

polo (AB TI) AND gerontology (Subdiscipline) AND Clinical trial (method)

**Results: 0**

power lifting (AB TI) AND gerontology (Subdiscipline) AND Clinical trial (method)

**Results: 5**

run* (AB TI) AND gerontology (Subdiscipline) AND Clinical trial (method)

**Results: 39**

rowing (AB TI) AND gerontology (Subdiscipline) AND Clinical trial (method)

**Results: 3**

sailing (AB TI) AND gerontology (Subdiscipline) AND Clinical trial (method)

**Results: 0**

shooting (AB TI) AND gerontology (Subdiscipline) AND Clinical trial (method)

**Results: 0**

skating (AB TI) AND gerontology (Subdiscipline) AND Clinical trial (method)

**Results: 0**

snowboard (AB TI) AND gerontology (Subdiscipline) AND Clinical trial (method)

**Results: 0**

soccer (AB TI) AND gerontology (Subdiscipline) AND Clinical trial (method)

**Results: 3**

surfing (AB TI) AND gerontology (Subdiscipline) AND Clinical trial (method)

**Results: 0**

swimming (AB TI) AND gerontology (Subdiscipline) AND Clinical trial (method)

**Results: 14**

table tennis (AB TI) AND gerontology (Subdiscipline) AND Clinical trial (method)

**Results: 1**

Taekwondo (AB TI) AND gerontology (Subdiscipline) AND Clinical trial (method)

**Results: 0**

Tae Kwon Do (AB TI) AND gerontology (Subdiscipline) AND Clinical trial (method)

**Results: 0**

tenpin bowling (AB TI) AND gerontology (Subdiscipline) AND Clinical trial (method)

**Results: 0**

tennis (AB TI) AND gerontology (Subdiscipline) AND Clinical trial (method)

**Results: 2**

trampoline (AB TI) AND gerontology (Subdiscipline) AND Clinical trial (method)

**Results: 3**

triathlon* (AB TI) AND gerontology (Subdiscipline) AND Clinical trial (method)

**Results: 0**

volleyball (AB TI) AND gerontology (Subdiscipline) AND Clinical trial (method)

# APPENDIX 3.

## Flow chart of selection of primary studies investigating the effects of physical activity programmes and services for older people (1)

Total number of primary study records: **(n=8,267)**

- Reviews included in the scoping review (n=350 reviews, 7,672 primary study records)
- Reviews identified in the updated search (n=25 reviews, 502 primary study records)

Duplicate records excluded / records excluded by title screening (n=5,616)



Eligible primary studies records included in *Component 1* **(n=1,421)**

- Records identified for full text review (n=2,651)
- *Additional records identified from sports search for full text review (n=21)*

Full-text records excluded: (n=1,251)

Reasons for exclusion:

- Population: younger (n=111)
- Population: disease groups (n=226)
- Population: other (n=6)
- Ineligible intervention (n=421)
- Ineligible outcome (n=100)
- Ineligible study design (n=198)
- Ineligible setting: hospital (n=12)

Records excluded (n=1,314)

- Study design (Non-RCT): (n=303)
- Average group size < 50: (n=821)
- PEDro score < 6 (n=586)
- Between-group comparisons results not reported: (n=5)

Eligible records included in *Component 2* **(n=107)**

Number of intervention groups (n=106)

Number of studies reported in the included records: (n=87)

**APPENDIX 4.**

## Eligibility criteria for selection of primary studies (1)

| **Category** | **Inclusion** | **Exclusion** |
| --- | --- | --- |
| Publication language | Full text in English | Abstract-only, other languages |
| Publication date | No limits | N/A |
| Publication status | Published in peer-reviewed journals | Unpublished studies |
| Study design | Evaluative studies (randomised-controlled trials, quasi-randomised trials, before and after studies) | - Cross-sectional studies - Case studies - Longitudinal exploratory studies (i.e. not investigating the effect of an intervention, but instead the association between variables) |
| **Characteristics of individual studies** | | |
| Population | - Adults 60+ years not selected on the basis of pre-existing conditions. Included studies may investigate samples of older adults who are: healthy; frail; with a disability, bone mineral density conditions, mild cognitive impairment, sarcopenia, obesity, mixed chronic conditions (e.g. diabetes, hypertension), or others. - Studies including younger participants where average age >=60 years | - Samples selected for pre-existing medical conditions (e.g. stroke, dementia, Parkinson’s, osteoporosis, hip fracture, depression, visual impairment osteoarthritis) - Studies including younger participants where the average age is <60 years |
| Intervention | Physical activity programs or services:   - “Whole body” Physical activity promoted or delivered - Supervised/unsupervised - All physical activity types - All physical activity providers - All program delivery modes | - Whole-body vibration /platform training - Combined interventions (e.g. supplement/nutrition and physical activity) - Occupational therapy/ physiotherapy without a clear physical activity component - Passive interventions, such as stretching. |
| Comparators | All included | NA |
| Outcomes | Any outcomes that measure physical activity, falls, physical function, participation/social, cognitive/emotional (mood) function, global measures (e.g. quality of life, frailty, functioning, wellbeing).  Physical function includes strength, fitness, balance, BMI, BMD, Cholesterol level, glucose level, | - Laboratory measures with no direct clinical use (inflammatory markers, neurotrophic biomarkers, lumbar spine kinematics, proprioception) or that are not routinely collected in clinical practice (e.g. Laboratory measures of cognitive function such as brain volume) - Joint-specific measures (e.g. Proprioception, ROM) - Adherence-only - Barriers & facilitators only - Fear of falling |
| Settings | Recruitment in all settings, but programs delivered in:   - Healthcare settings - Residential care - Community/home settings - Retirement villages | Programs delivered in inpatient settings |

BMI: body mass index, BMD: bone mineral density, ROM: range of motion

**APPENDIX 5.**

## Overview of primary studies of physical activity programs and services for older adults included in Objective 2 (k=87 studies) (1)

| **PICO/ TIDieR item** | **PICO aspect of interest, adaption of TIDieR** | **Framework classification** | | | **Number of studies in each category**  **k/87 (%)** | |
| --- | --- | --- | --- | --- | --- | --- |
| Population (Older adults) | Country | High-income | | | 81 (93%) | |
|  |  | Upper-middle income | | | 5 (6%) | |
|  |  | Lower-middle income | | | 0 | |
|  |  | Low income | | | 0 | |
|  |  | Mixed | | | 1 (1%) | |
|  | Remoteness | Rural/remote | | | 0 | |
|  |  | Urban | | | 13 (15%) | |
|  |  | Both | | | 1 (1%) | |
|  |  | Not-specified | | | 73 (84%) | |
|  | Age | Middle age (≥50 years) | | | 7 (8%) | |
|  |  | ≥60 years | | | 24 (28%) | |
|  |  | ≥65 years | | | 32 (37%) | |
|  |  | ≥70 | | | 17 (20%) | |
|  |  | ≥75 years | | | 3 (3%) | |
|  |  | ≥80 years | | | 3 (3%) | |
|  |  | ≥85 years | | | 1 (1%) | |
|  |  | ≥90 years | | | 0 | |
|  | Gender | Any | | | 76 (87%) | |
|  |  | Female | | | 8 (10%) | |
|  |  | Male | | | 3 (3%) | |
|  | Cultural /socio-economic background | Cultural | | | 0 | |
|  |  | Linguistic | | | 0 | |
|  |  | Migrant | | | 0 | |
|  |  | Indigenous | | | 0 | |
|  |  | Socio-economic | | | 0 | |
|  |  | No | | | 87 (100%) | |
|  | Living arrangements | Own home | | | 32 (37%) | |
|  |  | Public housing complex | | | 1 (1%) | |
|  |  | Retirement village | | | 4 (5%) | |
|  |  | Residential aged care facility | | | 9 (10 %) | |
|  |  | Not an eligibility criterion | | | 41 (47%) | |
|  | Physical activity level | Yes | | | 26 (30%) | |
|  |  | No | | | 61 (60%) | |
|  | Physical impairment/limitation | Mobility | | | 12 (14%) | |
|  |  | Frailty | | | 6 (7%) | |
|  |  | Osteopenia | | | 0 | |
|  |  | Sarcopenia | | | 0 | |
|  |  | Fall risk | | | 10 (11%) | |
|  |  | Nutrition | | | 0 | |
|  |  | Vision | | | 0 | |
|  |  | Hearing | | | 0 | |
|  |  | Pain | | | 0 | |
|  |  | Not an eligibility criterion | | | 59 (68%) | |
|  | Cognitive impairment or low mood | Cognitive impairment | Mild cognitive impairment | | 9 (10%) | |
|  |  | Low mood | Symptoms of depression | | 1 (1%) | |
|  |  |  | Symptoms of anxiety | | 0 | |
|  |  | Not an eligibility criterion | | | 77 (89%) | |
|  | Health conditions | Mixed chronic conditions | | | 3 (3%) | |
|  |  | Not an eligibility criterion | | | 84 (97%) | |
| Intervention* | Type of program | Physical activity delivered | | | 84 (79%) | |
|  |  | Physical activity promoted | Coaching | | 13 (12%) | |
|  |  |  | Referral | | 0 | |
|  |  |  | Brief/very brief intervention | | 3 (3%) | |
|  |  |  | eHealth/mHealth | | 1 (1%) | |
|  |  |  | Other | | 5 (5%) | |
|  | Type of physical activity | Overall activity | | | 21 (20%) | |
|  |  | Structured exercise | Balance, functional, neuromotor | | 3 (3%) | |
|  |  |  | Strength, resistance, power | | 14 (13%) | |
|  |  |  | Walking/wheeling | | 1 (1%) | |
|  |  |  | Endurance | | 3 (3%) | |
|  |  |  | High-intensity interval training | | 0 | |
|  |  |  | Multicomponent | | 38 (36%) | |
|  |  | Recreation | Tai Chi | | 16 (15%) | |
|  |  |  | Yoga/Pilates | | 3 (3%) | |
|  |  |  | Dance | | 5 (4%) | |
|  |  |  | Non-competitive sport | | 0 | |
|  |  |  | Other | | 0 | |
|  |  | Competitive sport | Standard | | 1 (1%) | |
|  |  |  | Modified | | 0 | |
|  |  | Exercise-based videogame | | | 1 (1%) | |
|  | Prescribed dose | Interventions | Supervised | | 97 (89%) | |
|  |  |  | Unsupervised | | 50 (46%) | |
|  |  | Session duration  *(minutes/session)* | Supervised ^ | | n=97  (91%) | Mean 49.2  SD (18.7) |
|  |  |  | Unsupervised ^ | | n=50  (47%) | Mean 38.7  SD (16.1) |
|  |  | Session frequency  *(time/week)* | Supervised^ | | n=95  (89%) | Mean 1.8  SD (1.2) |
|  |  |  | Unsupervised ^ | | n=50  (47%) | Mean 4.4  SD (2.2) |
|  |  | Program duration  *(weeks)* | Supervised ^ | | n=96  (90%) | Mean 36.6  SD (34.8) |
|  |  |  | Unsupervised^ | | n=50  (47%) | Mean 42.7  SD (34.1) |
|  | Type of supervision | In-person | | | 77 (72%) | |
|  |  | Online | | | 3 (3%) | |
|  |  | Not applicable | | | 22 (21%) | |
|  |  | Both | | | 4 (4%) | |
|  | Additional strategies to support physical activity^@^ | Behaviour change | Activity monitor | | 14 (13%) | |
|  |  |  | Mentoring | | 13 (12%) | |
|  |  |  | Social activity | | 6 (5%) | |
|  |  |  | Incentives | | 8 (7%) | |
|  |  |  | Booklet | | 9 (8%) | |
|  |  |  | Other | | 32 (30%) | |
|  |  | No additional strategies used | | | 58 (54%) | |
|  | Number of additional strategies to support physical activity per study | 1 | | | 21 (20%) | |
|  |  | 2 | | | 21 (20%) | |
|  |  | 3 | | | 7 (7%) | |
|  | Who prescribed | Professional | Health professional | | 61 (57%) | |
|  |  |  | Physical activity leader | | 45 (43%) | |
|  |  | Volunteer | No age restriction volunteer | | 0 | |
|  |  |  | Peer volunteer | | 0 | |
|  |  | Carer/caregiver | Professional carer/caregiver | | 0 | |
|  |  |  | Family/peer-support | | 0 | |
|  | Who delivered (if different to who prescribed) | Delivered by different personnel | | | 43 (40%) | |
|  |  | Delivered by same personnel | | | 40 (38%) | |
|  |  | Not applicable (e.g., home exercise program) | | | 23 (22%) | |
|  | Who with | Individual | | | 43 (41%) | |
|  |  | One other person | | | 2 (2%) | |
|  |  | Group | | | 47 (44%) | |
|  |  | Combination | | | 14 (13%) | |
|  | Delivery mode | Synchronous/ live | | In person | 81 (77%) | |
|  |  |  |  | Video | 0 | |
|  |  |  |  | Phone | 6 (6%) | |
|  |  |  |  | App/Web-based | 0 | |
|  |  | Asynchronous/ pre-recorded | | Video/DVD | 3 (3%) | |
|  |  |  |  | App/Web-based | 3 (3%) | |
|  |  |  |  | Exercise-based videogame | 1 (1%) | |
|  |  | Mixed | | | 11 (10%) | |
|  | Location^&^ | Community facility | | | 45 (42%) | |
|  |  | Own home | | | 24 (23%) | |
|  |  | Health facility | | | 4 (4%) | |
|  |  | Residential aged care facility | | | 11 (10%) | |
|  |  | Retirement village | | | 4 (4%) | |
|  | Equipment | Yes | | | 47 (44%) | |
|  |  | No | | | 59 (56%) | |
| Outcome^#^ | Physical activity | Steps | | | 6 (5%) | |
|  |  | Light physical activity | | | 0 | |
|  |  | Moderate-vigorous physical activity | | | 21 (16%) | |
|  |  | Up-time | | | 0 | |
|  |  | Overall physical activity | | | 28 (26%) | |
|  |  | Domains of physical activity | | | 14 (10%) | |
|  | Falls | Rate of falls | | | 34 (26%) | |
|  |  | Falls related injuries | | | 12 (9%) | |
|  | Intrinsic capacity: Physical domain | Bone mineral density | | | 8 (6%) | |
|  |  | Body composition | | | 14 (11%) | |
|  |  | Cardiometabolic indicators | | | 14 (11%) | |
|  |  | Strength | | | 35 (27%) | |
|  |  | Fitness | | | 13 (10%) | |
|  |  | Pain | | | 5 (4%) | |
|  |  | Other | | | 4 (4%) | |
|  | Functional ability: Physical domain | Mobility and balance | | | 67 (51%) | |
|  |  | Self-care | | | 13 (10%) | |
|  |  | Overall function | | | 24 (18%) | |
|  |  | Other | | | 5 (4%) | |
|  | Functional ability: Social domain | Self-report | | | 11 (8%) | |
|  |  | Observation | | | 0 | |
|  | Cognitive and emotional functioning | Cognitive | | | 25 (19%) | |
|  |  | Emotional | | | 19 (14%) | |
|  |  | Both | | | 7 (5%) | |
|  | Well-being and quality of life | Well-being | | | 0 | |
|  |  | Quality of life | | | 22 (17%) | |

* Percentages reported refer to the number of interventions (n=106) as some studies had more than two groups

^@^ The total percentage is more than 100% as individual interventions may involve more than one component

# Percentages refers to number of comparisons (n=131) not the number of studies (n=87)

^ Median and Standard Deviation (SD) only calculated for studies that reported the data

^&^  A simplified version of the location classification is presented int his table. Each study was classified according to its main location. Ccommunity centre includes public housing complex (n=1), workplace (n=0), faith-based facility (n=3), parks/sports fields (n=5) and research centre (n=15).

# APPENDIX 6.

## Behaviour change techniques and groupings by physical activity intervention

|  | Group 1  Goals and planning | | | | | | | Group 2  Feedback and monitoring | | | | | Group 3  Social  support | | | 4 | Group 5  Natural  Consequ-ences | | | Group 6  Compar-ison of behaviour | | | 7 | Group 8  Repetition and  substitution | | | | | | 9 | Group 10  Reward and  threat | | | | | 12 | Group 13  Identity | | 15 |  |
| --- | --- | --- | --- | --- | --- | --- | --- | --- | --- | --- | --- | --- | --- | --- | --- | --- | --- | --- | --- | --- | --- | --- | --- | --- | --- | --- | --- | --- | --- | --- | --- | --- | --- | --- | --- | --- | --- | --- | --- | --- |
| BCT no in group | **1** | **2** | **3** | **4** | **5** | **6** | **8** | **1** | **2** | **3** | **4** | **7** | **1** | **2** | **3** | **1** | **1** | **2** | **3** | **1** | **2** | **3** | **1** | **1** | **2** | **3** | **4** | **6** | **7** | **1** | **1** | **2** | **3** | **4** | **6** | **5** | **2** | **4** | **4** |  |
| Shimada et al 2018 |  |  |  | X |  |  |  |  |  |  |  |  |  |  |  | X | X |  |  | X |  |  |  | X |  |  |  |  |  | X |  |  |  |  |  |  |  |  |  | **6** |
| Hsieh et al 2019 |  |  |  | X | X |  |  |  |  | X |  |  | X |  |  | X |  |  |  |  |  |  |  | X |  |  |  |  | X | X |  |  |  |  |  | X |  |  |  | **9** |
| Lam et al 2014 |  |  |  | X |  |  |  |  |  |  |  |  |  |  |  | X |  |  |  | X |  |  |  | X |  |  |  | X |  |  |  |  |  |  |  |  |  |  |  | **5** |
| Morey et al 2009 | X | X |  | X | X |  |  |  | X |  |  |  | X |  |  | X | X |  |  | X |  |  |  |  |  |  |  |  |  | X |  |  |  |  |  | X |  |  |  | **11** |
| Thomas et al 2012 | X | X |  | X |  |  |  |  | X | X |  |  | X |  |  | X |  |  |  |  |  |  |  | X |  |  |  |  | X |  |  |  |  |  |  |  |  |  |  | **9** |
| Thomas et al 2012 | X | X |  | X |  |  |  |  | X | X |  |  |  |  |  | X |  |  |  |  |  |  |  | X |  |  |  |  | X |  |  |  |  |  |  |  |  |  |  | **8** |
| Thomas et al 2012 | X | X |  | X |  |  |  |  | X | X |  |  |  |  |  | X |  |  |  |  |  |  |  | X |  |  |  |  | X |  |  |  |  |  |  |  |  |  |  | **8** |
| Mantyet al 2009 |  | X |  | X | X | X | X |  |  |  |  |  | X | X |  | X | X |  |  | X |  |  |  | X | X |  | X |  |  | X |  |  |  |  |  |  |  |  |  | **14** |
| Rasinaho et al 2012 |  |  |  |  |  |  |  |  |  |  |  |  |  |  |  |  |  |  |  |  |  |  |  |  |  |  |  |  |  |  |  |  |  |  |  |  |  |  |  |  |
| von Bonsdorff et al 2008 |  |  |  |  |  |  |  |  |  |  |  |  |  |  |  |  |  |  |  |  |  |  |  |  |  |  |  |  |  |  |  |  |  |  |  |  |  |  |  |  |
| Kerse et al 2005 |  |  |  |  |  |  |  |  |  |  |  |  | X |  | X | X |  |  |  |  |  |  |  |  |  |  |  |  | X |  |  |  |  |  |  |  |  |  |  | **4** |
| Herghelegiu et al 2017 | X | X |  | X | X |  | X |  |  |  |  |  |  | X | X | X | X |  |  |  |  |  |  |  |  | X | X |  |  | X |  |  |  |  |  |  |  |  |  | **12** |
| McMurdo et al 2010 |  | X | X | X | X |  |  |  |  | X |  |  | X |  |  | X | X |  | X |  |  |  |  |  |  |  |  |  | X |  |  |  |  |  |  |  |  |  |  | **10** |
| McMurdo et al 2010 |  | X | X | X | X |  |  |  |  | X |  |  | X |  |  | X | X |  | X |  |  |  |  |  |  |  |  |  | X |  |  |  |  |  |  |  |  |  |  | **10** |
| Thomas et al 2005 |  |  |  | X |  |  |  |  |  |  |  |  |  |  |  | X |  |  |  | X |  |  |  | X |  |  |  |  |  |  |  |  |  |  |  |  |  |  |  | **4** |
| Thomas et al 2005 |  |  |  | X |  |  |  |  |  |  |  |  | X |  |  | X |  |  |  | X |  |  |  | X |  |  |  |  |  |  |  |  |  |  |  |  |  |  |  | **5** |
| Jette et al 1999 | X | X |  | X |  |  | X |  |  | X |  |  | X |  | X | X |  |  | X | X | X |  |  | X |  |  |  |  | X | X |  | X |  |  |  | X | X |  |  | **17** |
| King et al 2007 | X | X |  | X | X |  |  |  |  | X |  |  | X |  | X |  | X |  |  |  |  |  |  |  |  |  |  |  | X |  |  |  |  |  |  |  |  |  |  | **9** |
| King et al 2007 | X | X |  | X | X |  |  |  |  | X |  |  | X |  |  |  | X |  |  |  |  |  |  |  |  |  |  |  | X |  |  |  |  |  |  |  |  |  |  | **8** |
| Bogaerts et al 2009 |  |  |  | X |  |  |  |  |  |  |  |  |  |  |  | X |  |  |  | X |  |  |  | X |  |  |  |  | X |  |  |  |  |  |  |  |  |  |  | **5** |
| Bogaerts et al 2009 |  |  |  | X |  |  |  |  |  |  |  |  |  |  |  | X |  |  |  | X |  |  |  | X |  |  |  |  | X |  |  |  |  |  |  |  |  |  |  | **5** |
| Chin et al 2006 |  |  |  | X |  |  |  |  | X |  |  |  |  |  |  | X |  |  |  | X |  |  |  | X |  |  |  |  | X | X |  |  |  |  |  |  |  |  |  | **7** |
| Chin et al 2004 |  |  |  |  |  |  |  |  |  |  |  |  |  |  |  |  |  |  |  |  |  |  |  |  |  |  |  |  |  |  |  |  |  |  |  |  |  |  |  |  |
| Chin et al 2006 |  |  |  | X |  |  |  |  | X |  |  |  |  |  |  | X |  |  |  | X |  |  |  | X |  |  |  |  | X | X |  |  |  |  |  |  |  |  |  | **7** |
| Chin et al 2004 |  |  |  |  |  |  |  |  |  |  |  |  |  |  |  |  |  |  |  |  |  |  |  |  |  |  |  |  |  |  |  |  |  |  |  |  |  |  |  |  |
| Chin et al 2006 |  |  |  | X |  |  |  |  |  |  |  |  |  |  |  | X |  |  |  | X |  |  |  | X |  |  |  |  | X | X |  |  |  |  |  |  |  |  |  | **6** |
| Chin et al 2004 |  |  |  |  |  |  |  |  |  |  |  |  |  |  |  |  |  |  |  |  |  |  |  |  |  |  |  |  |  |  |  |  |  |  |  |  |  |  |  |  |
| Broekhuizen et al 2016 | X |  |  |  | X |  |  |  | X |  |  |  |  |  | X | X |  |  |  |  |  |  |  |  |  |  |  |  | X |  |  |  |  |  |  |  |  |  |  | **6** |
| Wijsman et al 2013 |  |  |  |  |  |  |  |  |  |  |  |  |  |  |  |  |  |  |  |  |  |  |  |  |  |  |  |  |  |  |  |  |  |  |  |  |  |  |  |  |
| Klusmann et al 2010 |  |  |  | X |  |  |  |  |  |  |  |  |  |  |  | X |  |  |  | X |  |  |  | X |  |  |  |  |  |  |  |  |  |  |  |  |  |  |  | **4** |
| Faber et al 2006 |  |  |  | X |  |  |  |  |  |  |  |  | X |  |  | X |  |  |  | X |  |  |  | X |  |  |  |  | X |  |  |  |  |  |  |  |  |  |  | **6** |
| Faber et al 2006 |  |  |  | X |  |  |  |  |  |  |  |  | X |  |  | X |  |  |  | X |  |  |  | X |  |  |  |  | X |  |  |  |  |  |  |  |  |  |  | **6** |
| Freiberger et al 2012 |  |  |  | X | X |  |  | X |  |  |  |  |  |  |  | X |  |  |  | X |  |  |  | X |  |  |  | X | X |  |  |  |  |  |  |  |  |  |  | **8** |
| Freiberger et al 2012 |  |  |  | X | X |  |  | X |  |  |  |  |  |  |  | X |  |  |  | X |  |  |  | X |  |  |  | X | X |  |  |  |  |  |  |  |  |  |  | **8** |
| Sun et al 2015 |  |  |  | X |  |  |  |  |  |  |  |  |  |  |  | X |  |  |  | X |  |  |  | X |  |  |  |  |  |  |  |  |  |  |  |  |  |  |  | **4** |
| Sherrington et al 2008 |  |  |  | X |  |  |  |  |  |  |  |  | X |  |  | X |  |  |  | X |  |  |  | X |  |  |  |  | X | X |  |  |  |  |  |  |  |  |  | **7** |
| Morey et al 2006 | X | X |  | X | X |  | X |  |  | X |  | X |  |  |  | X | X |  |  | X | X |  |  |  |  |  |  |  |  | X |  |  |  | X |  |  | X | X | X | **16** |
| Kolt et al 2007 | X | X |  | X |  | X |  |  |  | X |  |  | X | X | X | X | X |  |  |  |  |  | X |  |  |  |  |  | X |  |  |  | X |  |  |  |  |  |  | **13** |
| Yamada et al 2011 |  |  |  | X |  |  |  |  |  |  |  |  |  |  |  | X |  |  |  | X |  |  |  | X |  |  |  |  |  |  |  |  |  |  |  |  |  |  |  | **4** |
| Machacova et al 2017 |  |  |  | X |  |  |  |  |  |  |  |  |  |  |  | X |  |  |  | X |  |  |  | X |  |  |  |  | X |  |  |  |  |  |  |  |  |  |  | **5** |
| Maki et al 2012 | X |  |  | X | X |  |  |  |  | X |  |  | X | X |  |  |  |  |  |  |  |  |  |  |  |  |  | X | X |  |  |  |  |  |  |  |  |  |  | **8** |
| Gschwind et al 2015 |  |  |  | X |  |  |  |  |  | X |  |  | X |  |  | X | X |  |  | X |  |  | X |  |  |  |  |  | X |  |  |  |  |  |  |  |  |  |  | **8** |
| Best et al 2015 |  | X |  | X |  |  |  | X |  |  |  |  | X |  | X |  |  |  |  |  | X |  |  |  |  |  |  |  | X |  |  |  |  |  |  |  |  |  |  | **7** |
| Best et al 2015 |  | X |  | X |  |  |  | X |  |  |  |  | X |  | X |  |  |  |  |  | X |  |  |  |  |  |  |  | X |  |  |  |  |  |  |  |  |  |  | **7** |
| Lautenschlager et al 2008 | X | X |  | X |  |  |  |  |  | X |  |  | X |  |  | X |  |  |  | X |  |  |  |  |  |  |  |  | X |  |  |  | X |  |  |  |  |  |  | **9** |
| Vogler et al 2009 |  |  |  | X |  |  |  |  |  |  |  |  |  |  |  | X |  |  |  | X |  |  |  |  |  |  |  |  | X | X |  |  |  |  |  | X |  |  |  | **6** |
| Vogler et al 2009 |  |  |  | X |  |  |  |  |  |  |  |  |  |  |  | X |  |  |  | X |  |  |  |  |  |  |  |  | X | X |  |  |  |  |  | X |  |  |  | **6** |
| Mulrow et al 1994 |  |  |  | X |  |  |  |  |  |  |  |  |  |  |  | X |  |  |  |  |  |  |  |  |  |  |  |  | X | X |  |  |  |  |  | X |  |  |  | **5** |
| Wolf et al 1996 |  |  |  | X |  |  |  |  |  |  |  |  |  |  |  | X |  |  |  | X |  |  |  | X |  |  |  | X | X |  |  |  |  |  |  |  |  |  |  | **6** |
| Doi et al 2017 |  |  |  | X |  |  |  |  |  |  |  |  |  |  |  | X |  |  |  | X |  |  |  | X |  |  |  | X |  |  |  |  |  |  |  |  |  |  |  | **5** |
| Dubbert et al 2008 | X | X |  | X | X |  |  |  |  | X |  |  |  |  | X | X | X |  |  |  |  | X |  |  |  |  |  |  | X |  |  |  |  |  |  | X |  |  |  | **11** |
| Bernocchi et al 2019 |  |  |  | X |  |  |  |  | X | X | X |  | X | X |  | X | X |  |  |  |  |  |  |  |  |  |  |  | X | X |  |  |  |  |  | X |  |  |  | **11** |
| Petrella et al 2003 |  |  |  | X |  |  |  |  |  | X | X |  |  | X |  | X | X |  |  |  |  |  |  |  |  |  |  |  | X | X |  |  |  |  |  |  |  |  |  | **8** |
| Martin-Borras et al 2018 |  | X |  | X |  |  |  |  |  |  |  |  | X | X | X |  |  |  | X | X |  | X |  | X |  | X |  | X | X |  |  | X |  | X |  | X |  |  |  | **15** |
| Yates et al 2017 | X | X |  | X | X |  |  |  |  | X |  |  | X |  |  |  | X | X |  |  | X |  |  |  |  |  |  | X | X |  |  |  |  |  |  |  |  |  |  | **11** |
| Williamson et al 2009 |  |  |  | X |  |  |  |  |  |  |  |  | X | X |  | X | X |  |  | X |  |  |  |  |  |  | X | X | X | X |  |  |  |  |  | X |  |  |  | **11** |
| Pahor et al 2006 |  |  |  |  |  |  |  |  |  |  |  |  |  |  |  |  |  |  |  |  |  |  |  |  |  |  |  |  |  |  |  |  |  |  |  |  |  |  |  |  |
| Komulainen et al 2021 |  |  |  | X |  |  |  |  |  |  | X |  |  | X | X | X |  |  |  |  |  |  |  |  |  |  |  |  | X | X |  |  |  |  |  |  |  |  |  | **7** |
| Komulainen et al 2021 |  |  |  | X |  |  |  |  |  |  |  |  |  | X | X |  | X |  |  |  |  |  |  |  |  |  |  |  | X | X |  |  |  |  |  |  |  |  |  | **6** |
| Suzuki et al 2013 |  |  |  | X |  |  |  |  |  | X |  |  |  | X |  | X | X |  |  | X |  |  |  | X |  |  |  | X |  | X |  |  |  |  |  |  |  |  |  | **9** |
| Uemura et al 2012 |  |  |  |  |  |  |  |  |  |  |  |  |  |  |  |  |  |  |  |  |  |  |  |  |  |  |  |  |  |  |  |  |  |  |  |  |  |  |  |  |
| Chandler et al 1998 |  |  |  | X |  |  |  |  |  |  |  |  |  |  |  | X |  |  |  |  |  |  |  | X |  |  |  |  | X | X |  |  |  |  |  | X |  |  |  | **6** |
| King et al 2000 |  | X |  | X |  |  |  |  |  |  | X |  | X |  |  | X |  |  |  | X |  |  |  | X |  |  |  | X | X |  |  |  |  |  |  | X |  |  |  | **10** |
| Sparrow et al 2011 |  |  |  | X | X |  |  |  |  |  |  |  |  |  |  | X |  |  |  |  |  |  |  | X |  |  |  | X | X |  |  |  |  |  |  | X |  |  |  | **7** |
| Shimada et al 2018 |  |  |  | X |  |  |  |  |  | X |  |  | X |  |  | X |  |  |  | X |  |  |  | X |  |  |  | X |  | X |  |  |  |  |  |  |  |  |  | **8** |
| Aibar-Almazán et al 2019 |  |  |  | X |  |  |  |  |  |  |  |  |  |  |  | X |  |  |  | X |  |  |  | X |  |  |  |  | X |  |  |  |  |  |  |  |  |  |  | **5** |
| Greendale et al 2009 |  |  |  | X |  |  |  |  |  |  |  |  | X |  |  | X |  |  |  | X |  |  |  | X |  |  |  |  | X |  |  |  |  |  |  |  |  |  |  | **6** |
| Muscari et al 2010 |  |  |  | X |  |  |  |  |  |  |  |  | X |  |  | X |  |  |  | X |  |  |  | X |  |  |  |  | X |  |  |  |  |  |  |  |  |  |  | **6** |
| Song et al 2019 | X |  |  | X |  |  |  |  |  | X |  |  | X |  |  | X |  |  |  | X |  |  |  | X |  |  |  |  | X |  |  | X |  |  |  |  |  |  |  | **9** |
| Lazarou et al 2017 |  |  |  | X |  |  |  |  |  |  |  |  |  |  |  | X |  |  |  | X |  |  |  | X |  |  |  |  | X |  |  |  |  |  |  |  |  |  |  | **5** |
| Krebs et al 1998 |  | X |  | X |  |  |  |  |  |  |  |  | X |  |  | X |  |  |  | X |  |  |  | X |  |  |  |  | X | X |  |  |  |  |  | X |  |  |  | **9** |
| Tajik et al 2018 |  |  |  | X |  |  |  |  |  |  |  |  |  |  |  | X |  |  |  | X |  |  |  | X |  |  |  |  | X |  |  |  |  |  |  |  |  |  |  | **5** |
| Fan et al 2020 |  |  |  | X |  |  |  |  |  |  |  |  |  |  |  | X |  |  |  | X |  |  |  | X |  |  |  |  |  |  |  |  |  |  |  |  |  |  |  | **4** |
| Jette et al 1996 |  | X |  | X |  |  |  |  |  | X |  |  |  |  |  | X | X |  |  | X |  |  |  | X |  |  |  |  | X | X | X |  |  |  |  | X |  |  |  | **10** |
| Pandya et al 2020 |  |  |  | X |  |  |  | X |  |  |  |  |  |  |  | X |  |  |  | X |  |  |  | X |  |  |  | X |  | X | X |  |  |  |  |  |  |  |  | **8** |
| Barban et al 2017 |  |  |  | X |  |  |  |  |  |  |  |  |  |  |  |  |  |  |  |  |  |  |  |  |  |  |  |  | X |  |  |  |  |  |  |  |  |  |  | **2** |
| Barban et al 2017 |  |  |  | X |  |  |  |  |  |  |  |  |  |  |  |  |  |  |  |  |  |  |  |  |  |  |  |  | X |  |  |  |  |  |  |  |  |  |  | **2** |
| Tsang et al 2013 |  |  |  | X |  |  |  |  |  |  |  |  |  |  |  | X |  |  |  | X |  |  |  | X |  |  |  | X |  |  |  |  |  |  |  |  |  |  |  | **5** |
| Total BCTs | **16** | **22** | **2** | **68** | **16** | **2** | **4** | **5** | **8** | **22** | **4** | **1** | **30** | **11** | **12** | **60** | **19** | **1** | **4** | **44** | **5** | **2** | **2** | **43** | **1** | **2** | **3** | **15** | **53** | **25** | **1** | **2** | **2** | **2** | **1** | **15** | **2** | **1** | **1** | **529** |

**Note 1:** In table headings, 4= Group 4- Shaping knowledge, 7= Group 7- Associations, 9= Group 9 - Comparison of outcomes, 12=Group 12- Antecedents, 15=Group 15- Self-belief

**Note 2**: Three groupings were not included in this table as they had 0 BCTs. These were: Group 11: Regulation, Group 14: Scheduled consequences and Group 16: Covert learning. Within each grouping, only BCTs that were present were included in the table. Therefore only 39 of a possible total of 93 BCTs are included in the table.

**Note 3:** BCT number and grouping in this table corresponded to the BCT Taxonomy (v1) below (2):

| **Gro****uping and BCTs** | **Grouping and BCTs** | **Grouping and BCTs** |
| --- | --- | --- |
| **1. Goals and planning** | **6. Comparison of behaviour** | **12. Antecedents** |
| - 1. [Goal setting (behavior)](#_bookmark21)   2. [Problem solving](#_bookmark35)   3. [Goal setting (outcome)](#_bookmark0)   4. [Action planning](#_bookmark4)   5. [Review behavior goal(s)](#_bookmark19)  - 1. [Discrepancy between current](#_bookmark37) [behavior and goal](#_bookmark37)  - 1. [Review outcome goal(s)](#_bookmark0)   2. [Behavioral contract](#_bookmark10)   3. [Commitment](#_bookmark19) | - 1. [Demonstration of the](#_bookmark28) [behavior](#_bookmark28)   2. [Social comparison](#_bookmark44)   3. [Information about others’](#_bookmark50) [approval](#_bookmark50) | - 1. [Restructuring the physical](#_bookmark1) [environment](#_bookmark1)   2. [Restructuring the social](#_bookmark14) [environment](#_bookmark14)   3. [Avoidance/reducing exposure to](#_bookmark32) [cues for the behavior](#_bookmark32)   4. [Distraction](#_bookmark48)   5. [Adding objects to the](#_bookmark0) [environment](#_bookmark0)   6. [Body changes](#_bookmark8) |
|  | **7. Associations** |  |
|  | - 1. [Prompts/cues](#_bookmark1)   2. [Cue signalling reward](#_bookmark7)   3. [Reduce prompts/cues](#_bookmark20)   4. [Remove access to the](#_bookmark25) [reward](#_bookmark25)  - 1. [Remove aversive stimulus](#_bookmark33)   2. [Satiation](#_bookmark39)   3. [Exposure](#_bookmark47)  - 1. [Associative learning](#_bookmark0) |  |
| **2. Feedback and monitoring** |  | **13. Identity** |
| - 1. [Monitoring of behavior](#_bookmark0) [by others without](#_bookmark0) [feedback](#_bookmark0)   2. [Feedback on behaviour](#_bookmark0)  - 1. [Self-monitoring of](#_bookmark11) [behaviour](#_bookmark11)  - 1. [Self-monitoring of](#_bookmark31) [outcome(s) of behaviour](#_bookmark31)   2. [Monitoring of outcome(s)](#_bookmark0) [of behavior without](#_bookmark0) [feedback](#_bookmark0)   3. [Biofeedback](#_bookmark10)   4. [Feedback on outcome(s)](#_bookmark22) [of behavior](#_bookmark22) |  | - 1. [Identification of self as role](#_bookmark14) [model](#_bookmark14)   2. [Framing/reframing](#_bookmark30)   3. [Incompatible beliefs](#_bookmark40)   4. [Valued self-identify](#_bookmark47)   5. [Identity associated with changed](#_bookmark56) [behavior](#_bookmark56) |
|  | **8. Repetition and substitution** |  |
|  | - 1. [Behavioral](#_bookmark9)   [practice/rehearsal](#_bookmark9)   - 1. [Behavior substitution](#_bookmark23)   2. [Habit formation](#_bookmark29)   3. [Habit reversal](#_bookmark40)   4. [Overcorrection](#_bookmark45)   5. [Generalisation of target](#_bookmark51) [behavior](#_bookmark51)   6. [Graded tasks](#_bookmark0) |  |
|  |  | **14. Scheduled consequences** |
|  |  | - 1. [Behavior cost](#_bookmark1)   2. [Punishment](#_bookmark3)   3. [Remove reward](#_bookmark7)   4. [Reward approximation](#_bookmark17)   5. [Rewarding completion](#_bookmark26)   6. [Situation-specific reward](#_bookmark43)   7. [Reward incompatible behavior](#_bookmark53)   8. [Reward alternative behavior](#_bookmark0)   9. [Reduce reward frequency](#_bookmark5)  - 1. [Remove punishment](#_bookmark18) |
| **3. Social support** |  |  |
| - 1. [Social support (unspecified)](#_bookmark42)   2. [Social support (practical)](#_bookmark0)   3. [Social support (emotional)](#_bookmark13) | **9. Comparison of outcomes** |  |
|  | - 1. [Credible source](#_bookmark6)   2. [Pros and cons](#_bookmark32)  - 1. [Comparative imagining of](#_bookmark55) [future outcomes](#_bookmark55) |  |
| **4. Shaping knowledge** |  | **15. Self-belief** |
| - 1. [Instruction on how to](#_bookmark24) [perform the behavior](#_bookmark24)   2. [Information about](#_bookmark38) [Antecedents](#_bookmark38)  - 1. [Re-attribution](#_bookmark49)   2. [Behavioral experiments](#_bookmark0) |  | - 1. [Verbal persuasion about](#_bookmark27) [capability](#_bookmark27)   2. [Mental rehearsal of successful](#_bookmark33) [performance](#_bookmark33)   3. [Focus on past success](#_bookmark40)   4. [Self-talk](#_bookmark46) |
|  | **10. Reward and threat** |  |
|  | - 1. [Material incentive (behavior)](#_bookmark1)   2. [Material reward (behavior)](#_bookmark15)   3. [Non-specific reward](#_bookmark41)   4. [Social reward](#_bookmark0)   5. [Social incentive](#_bookmark13)   6. [Non-specific incentive](#_bookmark36)  - 1. [Self-incentive](#_bookmark0)  - 1. [Incentive (outcome)](#_bookmark12)   2. [Self-reward](#_bookmark34)   3. [Reward (outcome)](#_bookmark0)   4. [Future punishment](#_bookmark16) |  |
| **5. Natural consequences** |  | **16. Covert learning** |
| - 1. [Information about health](#_bookmark3) [consequences](#_bookmark3)   2. [Salience of consequences](#_bookmark22)   3. [Information about social and](#_bookmark38) [environmental consequences](#_bookmark38)   4. [Monitoring of emotional](#_bookmark57) [consequences](#_bookmark57)   5. [Anticipated regret](#_bookmark0)   6. [Information about emotional](#_bookmark5) [consequences](#_bookmark5) |  | - 1. [Imaginary punishment](#_bookmark54)   2. [Imaginary reward](#_bookmark0)   3. [Vicarious consequences](#_bookmark2) |
|  | **11. Regulation** |  |
|  | - 1. [Pharmacological support](#_bookmark24)   2. [Reduce negative emotions](#_bookmark37)   3. [Conserving mental resources](#_bookmark48)   4. [Paradoxical instructions](#_bookmark52) |  |

1. Pinheiro MD, Oliveira J, Tiedemann A, et al.: *Physical activity programmes and services for older adults: a review of effectiveness from primary studies. Report for the World Health Organization*, 2021.

2. Michie S, Richardson M, Johnston M, et al.: The behavior change technique taxonomy (v1) of 93 hierarchically clustered techniques: building an international consensus for the reporting of behavior change interventions. *Annals of Behavioral Medicine.* 2013, *46:*81-95.

# APPENDIX 7.

Table A.7.1 Intervention effects (compared with no intervention) by the most common BCTs: impact on different outcome domains

Table A.7.2 Intervention effects (compared with no intervention) by BCT grouping impact on different outcome domains

|  | **N of comparisons** | All outcomes | Physical Activity | Physical domain  (Intrinsic capacity) | Physical domain (Functional ability) | Social domain (ICF participation) | Cognitive & emotional functioning | Well-being & QoL |
| --- | --- | --- | --- | --- | --- | --- | --- | --- |
| **All BCT groups** | **N of comparisons** | **784** | **132** | **182** | **217** | **31** | **169** | **53** |
|  | % positive | 75% | 96% | 69% | 77% | 100% | 79% | 69% |
|  | % pos & sig | 48% | 71% | 27% | 39% | 62% | 37% | 45% |
| **Goals and planning (group 1)** | **N of comparisons** | **141** | **24** | **33** | **38** | **5** | **31** | **10** |
|  | % positive | 79% | 96% | 69% | 76% | 100% | 83% | 67% |
|  | % pos & sig | 41% | 70% | 26% | 39% | 63% | 43% | 42% |
| **Feedback and monitoring (group 2)** | **N of comparisons** | **75** | **15** | **17** | **20** | **2** | **14** | **7** |
|  | **% positive** | **38%** | **100%** | **76%** | **70%** | **100%** | **76%** | **78%** |
|  | % pos & sig | 38% | 76% | 20% | 40% | 33% | 32% | 56% |
| **Social support (group 3)** | **N of comparisons** | **84** | **19** | **17** | **21** | **4** | **16** | **7** |
|  | % positive | 83% | 96% | 69% | 93% | 100% | 76% | 78% |
|  | % pos & sig | 42% | 72% | 23% | 45% | 57% | 28% | 56% |
| **Sharing knowledge (group 4)** | **N of comparisons** | **122** | **20** | **30** | **36** | **5** | **25** | **6** |
|  | % positive | 79% | 100% | 73% | 76% | 100% | 81% | 57% |
|  | % pos & sig | 39% | 72% | 26% | 37% | 33% | 42% | 43% |
| **Natural consequences (group 5)** | **N of comparisons** | **43** | **15** | **5** | **10** | **2** | **7** | **4** |
|  | % positive | 82% | 95% | 59% | 85% | 100% | 74% | 80% |
|  | % pos & sig | 49% | 73% | 28% | 44% | 80% | 26% | 40% |
| **Comparison of behaviour (group 6)** | **N of comparisons** | **91** | **12** | **23** | **26** | **4** | **22** | **4** |
|  | % positive | 76% | 97% | 55% | 76% | 100% | 88% | 60% |
|  | % pos & sig | 39% | 58% | 26% | 34% | 71% | 50% | 20% |
| **Associations (group 7)** | **N of comparisons** | **2** | **1** | **0** | **1** | **0** | **0** | **0** |
|  | % positive | 100% | 100% | 0% | 100% | 0% | 0% | 0% |
|  | % pos & sig | 100% | 100% | 0% | 100% | 0% | 0% | 0% |
| **Repetition and substitution (group 8)** | **N of comparisons** | **127** | **9** | **33** | **38** | **5** | **32** | **10** |
|  | % positive | 79% | 96% | 70% | 76% | 100% | 82% | 67% |
|  | % pos & sig | 41% | 71% | 26% | 39% | 57% | 42% | 42% |
| **Comparison of outcomes (group 9)** | **N of comparisons** | **49** | **8** | **13** | **14** | **1** | **11** | **2** |
|  | % positive | 70% | 93% | 61% | 66% | 100% | 69% | 50% |
|  | % pos & sig | 36% | 68% | 34% | 32% | 50% | 24% | 50% |
| **Reward and threat (group 10)** | **N of comparisons** | **11** | **3** | **1** | **1** | **1** | **4** | **1** |
|  | % positive | 79% | 88% | 67% | 83% | 100% | 74% | 100% |
|  | % pos & sig | 51% | 63% | 50% | 50% | 100% | 37% | 100% |
| **Antecendents (group 12)** | **N of comparisons** | **35** | **5** | **9** | **11** | **2** | **6** | **2** |
|  | % positive | 80% | 93% | 88% | 86% | 100% | 54% | 50% |
|  | % pos & sig | 48% | 80% | 50% | 54% | 80% | 8% | 50% |
| **Identity (group 13)** | **N of comparisons** | **4** | **1** | **1** | **1** | **0** | **1** | **0** |
|  | % positive | 67% | 100% | 67% | 83% | 0% | 43% | 0% |
|  | % pos & sig | 29% | 0% | 50% | 50% | 0% | 0% | 0% |

**APPENDIX 8.**

**APPENDIX 9.**

Intervention effects by BCT grouping and outcome domains

This figure presents the effect direction and statistical significance for outcome domains across primary studies.

The numbers in each cell indicate the number of intervention groups. All intervention arms from the same study were included as a unique intervention group.

The size of the circle depicts the number of intervention groups such that the larger the circle, the greater the number of intervention groups. Large circles represent ≥20 intervention groups; medium circles represent 10-20 intervention groups; small circles represent <10 intervention groups.

Shades of colour depict the overall positive effect direction such that the darker the colour, the higher the proportion of positive effects across comparisons. No shading reflects <50% positive effect or negative effects (there are none in this figure).

*If 50 to 75% of total outcomes were reported as positive and statistically significant. For example, for the goals and physical activity outcomes circle, * indicates that 6-9/12 of the interventions tested which included the BCT goal setting had a positive and significant effect on physical activity outcome compared to their comparison no intervention groups.

**If >75% of total outcomes were reported as positive and statistically significant.

**APPENDIX 10.**

**Reference List of included studies** (1-59)

1. Shimada H, Lee S, Akishita M, et al.: Effects of golf training on cognition in older adults: a randomised controlled trial. *J Epidemiol Community Health.* 2018, *72*.

2. Hsieh TJ, Su SC, Chen CW, et al.: Individualized home-based exercise and nutrition interventions improve frailty in older adults: a randomized controlled trial. *Int J Behav Nutr Phys Act.* 2019, *16*.

3. Lam L, Chan WM, Kwok TC, Chiu HF: Effectiveness of Tai Chi in maintenance of cognitive and functional abilities in mild cognitive impairment: a randomised controlled trial. *Hong Kong Med J.* 2014, *20*.

4. Morey MC, Peterson MJ, Pieper CF, et al.: The Veterans Learning to Improve Fitness and Function in Elders Study: a randomized trial of primary care-based physical activity counseling for older men. *J Am Geriatr Soc.* 2009, *57*.

5. Thomas GN, Macfarlane DJ, Guo B, et al.: Health promotion in older Chinese: a 12-month cluster randomized controlled trial of pedometry and "peer support". *Med Sci Sports Exerc.* 2012, *44*.

6. Manty M, Heinonen A, Leinonen R, et al.: Long-term effect of physical activity counseling on mobility limitation among older people: a randomized controlled study. *J Gerontol A Biol Sci Med Sci.* 2009, *64*.

7. Rasinaho M, Hirvensalo M, Törmäkangas T, et al.: Effect of physical activity counseling on physical activity of older people in Finland (ISRCTN 07330512). *Health Promot Int.* 2012, *27*.

8. Bonsdorff MB, Leinonen R, Kujala UM, et al.: Effect of physical activity counseling on disability in older people: a 2-year randomized controlled trial. *J Am Geriatr Soc.* 2008, *56*.

9. Kerse N, Elley CR, Robinson E, Arroll B: Is physical activity counseling effective for older people? A cluster randomized, controlled trial in primary care. *J Am Geriatr Soc.* 2005, *53*.

10. Herghelegiu AM, Moser A PG, Born S, Wilhelm M, AE. S: Effects of health risk assessment and counselling on physical activity in older people: a pragmatic randomised trial. *PLoS ONE [Electronic Resource].* 2017, *12(7):e0181371*.

11. McMurdo ME, Sugden J, Argo I, et al.: Do pedometers increase physical activity in sedentary older women? A randomized controlled trial. *J Am Geriatr Soc.* 2010, *58*.

12. Thomas GN, Hong AW, Tomlinson B, et al.: Effects of Tai Chi and resistance training on cardiovascular risk factors in elderly Chinese subjects: a 12-month longitudinal, randomized, controlled intervention study. *Clin Endocrinol (Oxf).* 2005, *63*.

13. Jette AM, Lachman M, Giorgetti MM, et al.: Exercise–it's never too late: the strong-for-life program. *Am J Public Health.* 1999, *89*.

14. King AC, Friedman R, Marcus B, et al.: Ongoing physical activity advice by humans versus computers: the Community Health Advice by Telephone (CHAT) trial. *Health Psychol.* 2007, *26*.

15. Bogaerts AC, Delecluse C, Claessens AL, et al.: Effects of whole body vibration training on cardiorespiratory fitness and muscle strength in older individuals (a 1-year randomised controlled trial). *Age Ageing.* 2009, *38*.

16. Chin A Paw  MJM, van Poppel MNM, Twisk JWR, W. vM: Effects of resistance and all-round, functional training on quality of life, vitality and depression of older adults living in long-term care facilities: a 'randomized' controlled trial [ISRCTN87177281]. *BMC Geriatrics.* 2004, *4:5*.

17. Chin A Paw MJM, van Poppel MNM, Twisk JWR, W. vM: Once a week not enough, twice a week not feasible? A randomised controlled exercise trial in long-term care facilities [ISRCTN87177281]. *Patient Education & Counseling.* 2006, *63(1–2):205–14*.

18. Broekhuizen K, de Jelle G, Wijsman CA, et al.: An internet-based physical activity intervention to improve quality of life of inactive older adults: a randomized controlled trial. . *J Med Internet Res. .* 2016, *18(4):e74*.

19. Wijsman CA, Westendorp RG, Verhagen EA, et al.: Effects of a web-based intervention on physical activity and metabolism in older adults: randomized controlled trial. *J Med Internet Res.* 2013, *15(11):e233*.

20. Klusmann V Evers A, Schwarzer R, Schlattmann P, Reischies FM, Heuser I, et al. : Complex mental and physical activity in older women and cognitive performance: a 6-month randomized controlled trial. . *J Gerontol A Biol Sci Med Sci* 2010, *65:*680–688.

21. Faber MJ Bosscher R, Chin A Paw MJ, van Wieringen PC. : Effects of exercise programs on falls and mobility in frail and pre-frail older adults: A multicenter randomized controlled trial. . *Arch Phys Med Rehabil. .* 2006, *87:*885-896.

22. Freiberger E, Häberle L, Spirduso WW, GA. RZ: Long‐Term Effects of Three Multicomponent Exercise Interventions on Physical Performance and Fall‐Related Psychological Outcomes in Community‐Dwelling Older Adults: A Randomized Controlled Trial. . *J Am Geriatr Soc. .* 2012, *60:*437–446.

23. Sun J, Kanagawa K, Sasaki J, et al.: Tai chi improves cognitive and physical function in the elderly: a randomized controlled trial. *J Phys Ther Sci. 2015.* 2015, *27:*1467–1471.

24. Sherrington C, Pamphlett PI, Jacka JA, et al.: Group exercise can improve participants' mobility in an outpatient rehabilitation setting: a randomized controlled trial. . *Clin Rehabil. .* 2008, *22(6):*493–502.

25. Morey MC, Ekelund C, Pearson M, et al.: Project LIFE: a partnership to increase physical activity in elders with multiple chronic illnesses. *J Aging Phys Act.* 2006, *14*.

26. Yamada M, Aoyama T, Hikita Y, et al.: Effects of a DVD-based seated dual-task stepping exercise on the fall risk factors among community-dwelling elderly adults. *Telemed J E Health.* 2011, *17:*768-772.

27. Machacova K, Vankova H, Volicer L, Veleta P, I. H: Dance as Prevention of Late Life Functional Decline Among Nursing Home Residents. *J Appl Gerontol. .* 2017, *36(12):1453–70*.

28. Maki Y, Ura C, Yamaguchi T, et al.: Effects of intervention using a community-based walking program for prevention of mental decline: a randomized controlled trial. *J Am Geriatr Soc.* 2012, *60*.

29. Gschwind YJ Eichberg S, Ejupi A, de Rosario H, Kroll M, Marston HR, et al. : ICT-based system to predict and prevent falls (iStoppFalls): results from an international multicenter randomized controlled trial. . *Eur Rev Aging Phys Act. .* 2015, *2:10*.

30. Best JR, Chiu BK, Liang Hsu C, Nagamatsu LS, Liu-Ambrose T: Long-Term Effects of Resistance Exercise Training on Cognition and Brain Volume in Older Women: Results from a Randomized Controlled Trial. *J Int Neuropsychol Soc.* 2015, *21*.

31. Lautenschlager NT, Cox KL, Flicker L, et al.: Effect of physical activity on cognitive function in older adults at risk for Alzheimer disease: a randomized trial. *JAMA.* 2008, *300*.

32. Vogler CM, Sherrington C, Ogle SJ, Lord SR: Reducing risk of falling in older people discharged from hospital: a randomized controlled trial comparing seated exercises, weight-bearing exercises, and social visits. *Arch Phys Med Rehabil.* 2009, *90*.

33. Mulrow CD, Gerety MB, Kanten D, et al.: A randomized trial of physical rehabilitation for very frail nursing home residents. Jama. . *JAMA.* 1994, *271(7):519–24*.

34. Wolf SL Barnhart H, Kutner NG, McNeely E, Coogler C, Xu T. : Reducing frailty and falls in older persons: an investigation of Tai Chi and computerized balance training. Atlanta FICSIT Group. Frailty and Injuries: Cooperative Studies of Intervention Techniques. . *J Am Geriatr Soc. .* 1996, *44(5):489–97*.

35. Doi T, Verghese J, Makizako H, et al.: Effects of Cognitive Leisure Activity on Cognition in Mild Cognitive Impairment: Results of a Randomized Controlled Trial. . *J Am Med Dir Assoc. .* 2017, *18(8):686–91*.

36. Dubbert PM, Morey MC, Kirchner KA, Meydrech EF, Grothe K: Counseling for home-based walking and strength exercise in older primary care patients. *Arch Intern Med.* 2008, *168*.

37. Bernocchi P, Giordano A, Pintavalle G, et al.: Feasibility and Clinical Efficacy of a Multidisciplinary Home-Telehealth Program to Prevent Falls in Older Adults: A Randomized Controlled Trial. . *J Am Med Dir Assoc. .* 2019, *20(3):340–6*.

38. Petrella RJ, Koval JJ, Cunningham DA, Paterson DH: Can primary care doctors prescribe exercise to improve fitness? The step test exercise prescription (STEP) project. *Am J Prev Med.* 2003, *24*.

39. Martin-Borras C, Giné-Garriga M, Puig-Ribera A, et al.: A new model of exercise referral scheme in primary care: is the effect on adherence to physical activity sustainable in the long term? A 15-month randomised controlled trial. *BMJ open.* 2018, *8(3):e017211*.

40. Yates T, Edwardson CL, Henson J, et al.: Walking Away from Type 2 diabetes: a cluster randomized controlled trial. *Diabet Med.* 2017, *34*.

41. Williamson JD Espeland M, Kritchevsky SB, Newman AB, King AC, Pahor M, et al. : Changes in cognitive function in a randomized trial of physical activity: results of the lifestyle interventions and independence for elders pilot study. *J Gerontol A Biol Sci Med Sci. .* 2009, *64(6):688–94*.

42. Pahor M, Guralnik JM, Ambrosius WT, et al.: Life study investigators, Effect of structured physical activity on prevention of major mobility disability in older adults: the LIFE study randomized clinical trial. *JAMA.* 2014, *311(23):2387–96*.

43. Komulainen P: Exercise, diet, and cognition in a 4-year randomized controlled trial: Dose-Responses to Exercise Training (DR’s EXTRA). *Am J Clin Nutr.* 2021, *113*.

44. Suzuki T, Shimada H, Makizako H, et al.: A randomized controlled trial of multicomponent exercise in older adults with mild cognitive impairment. *8(4):e61483.* 2013.

45. Uemura K Doi T, Shimada H, Makizako H, Yoshida D, Tsutsumimoto K, et al. : Effects of exercise intervention on vascular risk factors in older adults with mild cognitive impairment: a randomized controlled trial. . *Dement Geriatr Cogn Dis Extra.* 2012, *2(1):445–55*.

46. Chandler JM, Duncan PW, Kochersberger G, Studenski S: Is lower extremity strength gain associated with improvement in physical performance and disability in frail, community-dwelling elders? *Arch Phys Med Rehabil.* 1998, *79*.

47. Sparrow D, Gottlieb DJ, Demolles D, Fielding RA: Increases in muscle strength and balance using a resistance training program administered via a telecommunications system in older adults. *J Gerontol A Biol Sci Med Sci.* 2011, *66*.

48. Aibar-Almazán A M-Amat A, Cruz-Díaz D, De la Torre-Cruz MJ, Jiménez-García JD, Zagalaz-Anula N, et al. : Effects of Pilates on fall risk factors in community-dwelling elderly women: A randomized, controlled trial. *Eur J Sport Sci. .* 2019, *19(10):1386–94*.

49. Greendale GA Huang M, Karlamangla AS, Seeger L, Crawford S. : Yoga decreases kyphosis in senior women and men with adult-onset hyperkyphosis: results of a randomized controlled trial. . *J Am Geriatr Soc. .* 2009, *57(9):1569–79*.

50. Muscari A, Giannoni C, Pierpaoli L, et al.: Chronic endurance exercise training prevents aging-related cognitive decline in healthy older adults: a randomized controlled trial. *Int J Geriatr Psychiatry.* 2010, *25*.

51. Song D, Yu DSF: Effects of a moderate-intensity aerobic exercise program on the cognitive function and quality of life of community-dwelling elderly people with mild cognitive impairment: A randomised controlled trial. *Int J Nurs Stud.* 2019, *93*.

52. Lazarou I, Parastatidis T, Tsolaki A, et al.: International Ballroom Dancing Against Neurodegeneration: A Randomized Controlled Trial in Greek Community-Dwelling Elders With Mild Cognitive impairment. . *Am J Alzheimers Dis Other Demen.* 2017, *32(8):489–99*.

53. Krebs DE, Jette AM, Assmann SF: Moderate exercise improves gait stability in disabled elders. *Arch Phys Med Rehabil.* 1998, *79*.

54. Tajik A Rejeh N, Heravi-Karimooi M, Samady Kia P, Tadrisi SD, Watts TE, et al. : The effect of Tai Chi on quality of life in male older people: A randomized controlled clinical trial. . *Complement Ther Clin Pract. .* 2018, *33:191–6*.

55. Fan B, Song W, Zhang J, et al.: The efficacy of mind-body (Baduanjin) exercise on self-reported sleep quality and quality of life in elderly subjects with sleep disturbances: a randomized controlled trial. *Sleep Breath.* 2020, *24*.

56. Jette AM, Harris BA, Sleeper L, et al.: A home-based exercise program for nondisabled older adults. *J Am Geriatr Soc.* 1996, *44*.

57. Pandya SP: Yoga Education Program for Improving Memory in Older Adults: A Multicity 5-Year Follow-Up Study. *J Appl Gerontol.* 2020, *39*.

58. Barban F, Annicchiarico R, Melideo M, et al.: Reducing Fall Risk with Combined Motor and Cognitive Training in Elderly Fallers. *Brain Sci.* 2017, *7(2)*.

59. Tsang HW LJ, Au DW, Wong KK, Lai KW.: Developing and testing the effectiveness of a novel health qigong for frail elders in Hong Kong: a preliminary study. *Evid Based Complement Alternat Med. .* 2013, *2013:827392*.
